# Changes in DNA methylation contribute to rapid adaptation in bacterial plant pathogen evolution

**DOI:** 10.1371/journal.pbio.3002792

**Published:** 2024-09-20

**Authors:** Rekha Gopalan-Nair, Aurore Coissac, Ludovic Legrand, Céline Lopez-Roques, Yann Pécrix, Céline Vandecasteele, Olivier Bouchez, Xavier Barlet, Anne Lanois, Alain Givaudan, Julien Brillard, Stéphane Genin, Alice Guidot

**Affiliations:** 1 LIPME, Université de Toulouse, INRAE, CNRS, Castanet-Tolosan, France; 2 GeT-PlaGe, Genotoul, INRAE, US1426, Castanet-Tolosan, France; 3 PVBMT, Université de La Réunion, CIRAD, Saint-Pierre, Réunion Island, France; 4 DGIMI, Université de Montpellier, INRAE, Montpellier, France; Sainsbury Laboratory, UNITED KINGDOM OF GREAT BRITAIN AND NORTHERN IRELAND

## Abstract

Adaptation is usually explained by beneficial genetic mutations that are transmitted from parents to offspring and become fixed in the adapted population. However, genetic mutation analysis alone is not sufficient to fully explain the adaptive processes, and several studies report the existence of nongenetic (or epigenetic) inheritance that can enable adaptation to new environments. In the present work, we tested the hypothesis of the role of DNA methylation, a form of epigenetic modification, in adaptation of the plant pathogen *Ralstonia pseudosolanacearum* to the host during experimental evolution. Using SMRT-seq technology, we analyzed the methylomes of 31 experimentally evolved clones obtained after serial passages on 5 different plant species during 300 generations. Comparison with the methylome of the ancestral clone revealed a list of 50 differential methylated sites (DMSs) at the GTWWAC motif. Gene expression analysis of the 39 genes targeted by these DMSs revealed limited correlation between differential methylation and differential expression of the corresponding genes. Only 1 gene showed a correlation, the RSp0338 gene encoding the EpsR regulator protein. The MSRE-qPCR technology, used as an alternative approach for DNA methylation analysis, also found the 2 DMSs upstream RSp0338. Using site-directed mutagenesis, we demonstrated the contribution of these 2 DMSs in host adaptation. As these DMSs appeared very early in the experimental evolution, we hypothesize that such fast epigenetic changes can allow rapid adaptation to the plant stem environment. In addition, we found that the change in DNA methylation upstream RSp0338 remains stable at least for 100 generations outside the host and thus can contribute to long-term adaptation to the host plant. To our knowledge, this is the first study showing a direct link between bacterial epigenetic variation and adaptation to a new environment.

## Abstract 2

Do epigenetic changes contribute to long-term bacterial adaptation? This study shows that changes in DNA methylation enable rapid adaptation of the major agricultural bacterial pathogen Ralstonia pseudosolanacearum to novel hosts, stable over 100 generations.

## Introduction

Faced with the selection pressure imposed by their environment, pathogens must continuously adapt to survive and multiply. Many works aim to better understand the adaptive processes of pathogens in order to better apprehend the sustainability of the control strategies. Adaptation, the modification of the phenotype as a result of natural selection, is usually explained by beneficial genetic mutations that are transmitted from parents to offspring and become fixed in the adapted population [1–3]. However, more and more studies show that genetic mutation analysis alone is not sufficient to fully explain the processes of adaptive evolution and report the role of nongenetic (or epigenetic) inheritance in the generation of adapted phenotypes [4,5]. Models suggest that epigenetic inheritance of the parental phenotype can be adaptive in slowly fluctuating and correlated environments, since the parent and offspring will most often share the same environmental conditions [6,7]. However, direct tests of this prediction are, so far, lacking. Epigenetic changes were described to be more involved in short-term adaptation, or acclimation, by inducing phenotypic plasticity [8]. This was supported by the observation that epigenetic changes occur at a faster rate than genetic mutations but may be less stable [9,10]. However, recent works also support the hypothesis that epigenetic modifications could impact long-term adaptive responses to changing environments through the transgenerational inheritance of epigenetic signatures [5,8,10–13].

A well-documented epigenetic mechanism known to be involved in the modification of the phenotype is DNA methylation. DNA methylation consists in the addition of a methyl group (CH_3_) on the adenine or cytosine base of DNA catalyzed by DNA methyltransferases (MTases) that recognize specific DNA motifs. In bacterial genomes, methylated DNA is found in the forms of 6mA (6-methyladenine), which is the most prevalent form, 4mC (4-methylcytosine), and 5mC (5-methylcytosine) [14,15]. Many works demonstrated the role of DNA methylation in the regulation of important cellular functions in bacteria, including DNA replication, DNA repair, chromosome segregation, transcriptional regulation, phenotypic heterogeneity, and virulence [16–22]. Nowadays, thanks to the Pacbio sequencing technology, which enables the sequencing of single molecules in real time (SMRT-seq) without amplification, it is possible to analyze the 6mA and 4mC methylation profile of bacteria [14,15,23–27]. Here, we used SMRT-seq technology to explore the DNA methylation profile (methylome) of the model bacterial plant pathogen *Ralstonia pseudosolanacearum*. The purpose of this study was to test the hypothesis of methylome variation during the experimental adaptation of the bacteria to various host plants and investigate the potential role of methylome changes in the generation of adapted phenotypes.

*R*. *pseudosolanacearum* is part of the *Ralstonia solanacearum* species complex (RSSC), a soil-born plant pathogen responsible of the lethal bacterial wilt disease on more than 250 plant species including economically important crops such as tomato, potato, or banana [28]. This bacterium is worldwide distributed and represents a major threat in agriculture. It is characterized by a strong adaptive capacity, with no effective control method available today, and new strains capable of colonizing new hosts are continuously emerging [29–33]. Numerous works have been conducted with the aim of better understanding adaptive processes in RSSC. The role of genetic modifications of the bacterial genome such as mutation, transposable elements (TEs) movement, recombination, or horizontal gene transfer were reported [34–37]. However, the contribution of epigenetic modifications in RSSC adaptation has not yet been addressed.

A recent study compared the methylomes using SMRT-seq of 2 RSSC strains belonging to distant phylogenetic groups, the GMI1000 strain from phylotype I (*R*. *pseudosolanacearum*) and the UY031 strain from phylotype II (*R*. *solanacearum*) [38]. This work identified a commonly methylated motif in the 2 strains, the GTWWAC motif, 6mA methylated, associated with an MTase, M.RsoORF1982P, that is conserved in all RSSC genomes and across the Burkholderiaceae [38]. Analysis of the methylated regions in RSSC genomes identified genes involved in global and virulence regulatory functions, thus suggesting a role for DNA methylation in regulation of their expression.

In our previous works, we conducted an experimental evolution of the *R*. *pseudosolanacearum* GMI1000 strain in order to better understand the molecular bases of adaptation. In this experiment, strain GMI1000 was maintained in a fixed plant line during 300 generations by serial passages from stem to stem. This experiment was conducted on 6 different plant species including susceptible hosts (tomato var. Marmande, eggplant var. Zebrina, pelargonium var. Maverick Ecarlate) and tolerant hosts (bean var. Blanc Précoce, cabbage var. Bartolo, tomato var. Hawaii 7996) [37,39]. Most of the evolved clones showed a better fitness (better growth rate) in their experimental host than the ancestral clone. Whole genome sequence analysis revealed between 0 and 3 mutations in the adapted clones, and the role of some mutations in host adaptation was demonstrated [37,39–41]. However, in several adapted clones, no mutation could be detected, suggesting that epigenetic modifications may play a role in host adaptation. In addition, transcriptomic analysis of these clones revealed important differential gene expression compared to the ancestral clone, thus reinforcing the hypothesis of a role of epigenetic modification in gene expression change [39,42].

In this study, we analyzed the methylomes of 31 experimentally evolved clones using SMRT-seq. Comparison with the methylome of the ancestral GMI1000 clone revealed differential methylated sites (DMSs) at the GTWWAC motif in the evolved clones. Using site-directed mutagenesis, we demonstrated the contribution of 1 DMS in host adaptation, which, interestingly, turns out to be linked to a gene involved in the expression of a bacterial virulence determinant.

## Results

### Defining the methylation profile of strain GMI1000

In order to detect potential changes in the methylation profile of evolved clones, we first established the methylated motifs in the wild-type ancestor GMI1000 using SMRT-seq technology. In order to limit the number of cells in division and avoid a bias towards hemimethylated marks, genomic DNA was prepared from bacterial cells collected at the beginning of stationary phase. Growth was performed in synthetic medium with glutamine to mimic xylem environment of the plant, glutamine being the main compound of xylem sap in most plant species [43].

The global analysis of all modification marks on the GMI1000 DNA identified a total number of 45,831 modification marks above default thresholds. This number was much lower than that found in our previous work that reported 229,207 modification marks [38]. This difference probably results from several factors such as changing SMRT-seq technology and analysis pipelines. In the present study, 2 methylated motifs were detected in the GMI1000 genome, GTWWAC and YGCCGGCR. As the YGCCGGCR motif was detected with a very low percentage of methylation, although the sequencing depth was very high (160×), it suggested that this motif is associated to 5mC modification, which is difficult to detect by SMRT-seq. The third motif reported previously, C**C**CAKNAVCR [38], was not detected in the present work. As this motif was very degenerate and detected with a very weak signal, it was probably a false positive detection. For the comparative methylation analysis using SMRT-seq technology, we thus investigated the methylation profile of the GTWWAC motif in the ancestral and evolved clones.

**Fig 1:**
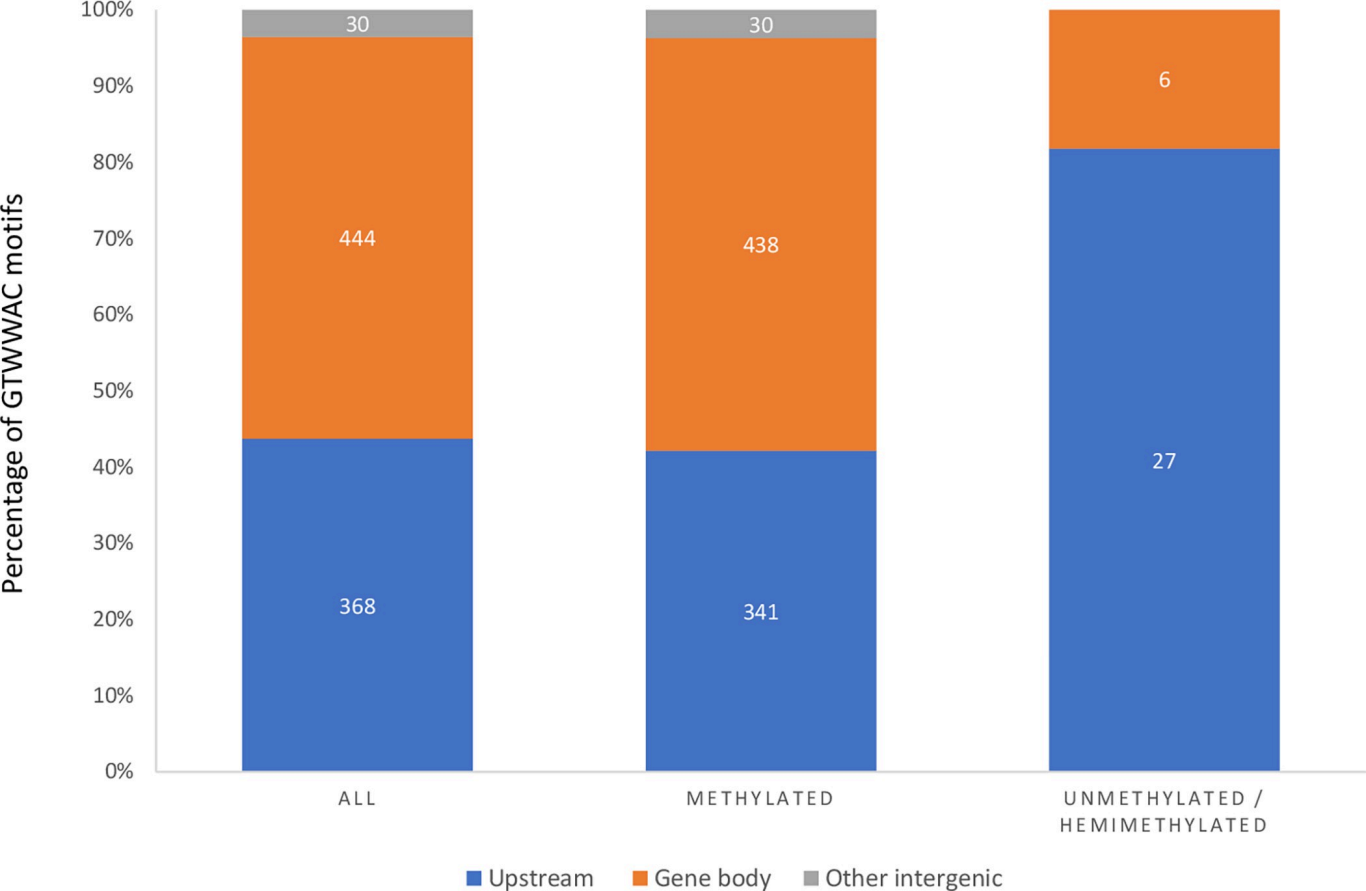
Distribution of the methylated and unmethylated GTWWAC motifs in the GMI1000 genome. Numbers and proportions of GTWWAC motifs located in gene body (blue), in putative promoter region (i.e., <300 bp upstream from a start codon) (orange) or in intergenic region (grey). When the same GTWWAC motif affects 2 genes, it was counted twice. The data underlying this figure can be found in S1 Data.

A total of 392 GTWWAC motifs are present in the GMI1000 genome and affect 366 genes either in the promoter region (i.e., <300 bp upstream from a start codon) or in the gene ORF, thus affecting 7% of all GMI1000 genes, whose number has been estimated at 5,129 [44]. In our culture and growth phase conditions and according to SMRT-seq data, 10 GTWWAC motifs were detected unmethylated and 9 motifs were hemimethylated (DNA methylation of either strand–or strand +) in the GMI1000 genome (Tables 1 and S1). The analysis of the distribution of methylated and unmethylated GTWWAC motifs with respect to genes or putative promoter regions showed that most (82%) of the unmethylated GTWWAC motifs were located in putative promoter regions, while only 42% of the methylated motifs were located in these regions (Fig 1). These unmethylated sites could be associated to potential regulatory regions where a competition between the MTase and a DNA binding protein could occur [45]. These sites specifically concerned the RSc0958 gene encoding a type VI secretion system tip VgrG family protein [46], the *epsR* gene (2 motifs) encoding the negative regulator of exopolysaccharide (EPS) production [47] and the *efe* gene encoding the ethylene-forming enzyme [48] (Table 1).

**Table 1:** Genomic regions of the GMI1000 strain of *R*. *pseudosolanacearum* with a GTWWAC motif detected unmethylated or hemimethylated at the beginning of the stationary phase during growth in synthetic medium with glutamine, according to SMRT-seq data.

Replicon	Gene ID	Gene name	Gene Description	position strand −	position strand +	Motif	upstream, intragenic*	methylation status**
								SMRT-seq
Chromosome	RSc0958		type VI secretion system tip VgrG family protein	1004576	1004579	GTTAAC	upstream	unmethylated
Chromosome	RSc2561		Conserved protein, DUF3313 domain-containing	2769503	2769506	GTTTAC	upstream	unmethylated
Chromosome	RSc2612		ICE Tn4371—Hypothetical protein	2813720	2813723	GTTTAC	intragenic	unmethylated
Chromosome	RSc3132		Transcription regulator, XRE family with a cupin C-terminal domain	3378821	3378824	GTTTAC	upstream	unmethylated
Megaplasmid	RSp0338	*epsR*	Negative regulator of EPS production EpsR, Transcription regulator, NarL/FixJ family	445723	445726	GTTTAC	upstream	unmethylated
Megaplasmid	RSp0338	*epsR*	Negative regulator of EPS production EpsR, Transcription regulator, NarL/FixJ family	445735	445738	GTAAAC	upstream	unmethylated
Megaplasmid	RSp0629		Type VI secretion system tip VgrG family protein with DUF2345 domain	765405	765408	GTTAAC	upstream	unmethylated
Megaplasmid	RSp1329		hypothetical protein	1680220	1680223	GTATAC	intragenic	unmethylated
Megaplasmid	RSp1398/RSp1399	*aroE2/*	shikimate 5-dehydrogenase/porin	1761411	1761414	GTAAAC	upstream	unmethylated
Megaplasmid	RSp1529	*efe*	1-aminocyclopropane-1-carboxylate oxidase (Ethylene-forming enzyme)	1916009	1916012	GTTTAC	upstream	unmethylated
Chromosome	RSc0081		Transcription regulator, MurR/RpiR family	94117	94120	GTTAAC	upstream	hemimethylated strand −
Chromosome	RSc0608	*ripAA*	type III effector protein RipAA	655714	655717	GTTAAC	upstream	hemimethylated strand −
Chromosome	RSc2094/RSc2095	*xanR/xdhA*	Purine salvage pathway regulator XanR, Transcription Regulator, LysR family/Xanthine Dehydrogenase, subunit A	2267247	2267250	GTTTAC	upstream	hemimethylated strand −
Megaplasmid	RSp1025		Translocator, LysE family	1298046	1298049	GTTTAC	upstream	hemimethylated strand −
Chromosome	RSc2176	*tISRso5*	ISRSO5-transposase protein	2360129	2360132	GTTAAC	upstream	hemimethylated strand +
Chromosome	RSc2176	*tISRso5*	ISRSO5-transposase protein	2360143	2360146	GTAAAC	upstream	hemimethylated strand +
Megaplasmid	RSp0216/RSp0217	*/ tISRso5*	Pseudogene: Type 3 Secretion effector RipBM (C-terminal fragment)/ ISRSO5-transposase protein	269725	269728	GTTAAC	intragenic / upstream	hemimethylated strand +
Megaplasmid	RSp1544		hypothetical protein	1939052	1939055	GTAAAC	intragenic	hemimethylated strand +
Megaplasmid	RSp1675	*tISRso5*	ISRSO5-transposase protein	2087332	2087335	GTTAAC	upstream	hemimethylated strand +

Note: Raw data from SMRT-seq analysis are given in S2 Table.

*GTWWAC motifs were annotated intragenic if their positions mapped within the annotated coding sequence and upstream if they mapped to the first noncoding 300 bp before the annotated start codon.

**For hemimethylated motifs, the strand which is methylated is indicated.

### Mapping differentially methylated sites between the ancestral and evolved clones with SMRT-sequencing

A total of 31 evolved clones derived from strain GMI1000 after experimental evolution in 5 different host plants over 300 generations were investigated (Table 2). Phenotypic and genotypic analyses of these clones were conducted in previous works [37,39,42]. These 31 evolved clones did not present any difference with the ancestral clone in terms of symptoms on plants, but most of them displayed a better multiplication rate in the plant xylem, as revealed by bacterial competition assays (see Materials and methods). In planta competition experiments between the ancestral and derived clones were conducted and a competitive index (CI) was calculated and used as a fitness estimator. All clones but one exhibited a better fitness than their ancestral clone in their experimental host (CI > 1) (Table 2). Only the clone Zeb26d1 recovered from eggplant Zebrina had a CI not significantly different from one and was used as a control. Genotypic analyses of the 31 evolved clones revealed an average of 1.2 (min 0; max 3) genomic polymorphisms in these clones [42] (Table 2).

**Table 2:** Investigated evolved clones (derived from [42]).

Experimental host	Lineage	Evolved clone	Mean CI ± SE (no. of replicates)	Mutations			no. of DEGs (I logFC I > 1; p-value FDR <0.05)	no. of DMSs at the GTWWAC motif
Tomato var. Marmande	A	Mar26a1	5.6 ± 0.9 (11)	RSc2508^IS, -120^	*tktA* ^R326G^	RSp0128-0154^Del 33kb^	1390	14
	A	Mar26a2	5.4 ± 1.4 (8)	RSc2508^IS, -120^			1424	13
	B	Mar26b2	3.9 ± 0.9 (8)	*phcS* ^T26M^			2368	19
	D	Mar26d2	5.7 ± 1.3 (13)	RSc2508^IS, -120^			1387	16
	E	Mar26e1	3.4 ± 0.5 (13)	RSc2508^IS, -120^			2174	16
	E	Mar26e3	6.3 ± 2.0 (11)	RSc2508^IS, -120^	RSp1466^In 8 nt, -256^		1371	16
Eggplant var. Zebrina	B	Zeb26b1	2.7 ± 0.4 (8)	RSp0083 ^IS, 1^			332	14
	B	Zeb26b5	3.7 ± 0.6 (7)				239	16
	C	Zeb26c2	2.1 ± 0.2 (9)	RSp0127^F91L^			92	15
	C	Zeb26c3	1.6 ± 0.2 (8)	RSp0127^F91L^			335	15
	C	Zeb26c4	2.1 ± 0.3 (8)	*dld* ^R135S^			25	14
	D	Zeb26d1	0.9^ns^ ± 0.1 (7)				353	16
	E	Zeb26e1	3.6 ± 1.0 (9)				515	17
Bean var. Blanc Précoce	A	Bean26a4	6.1 ± 1.0 (15)	RSc2508^A394(-)*^	*rpoB* ^D428Y^		1952	17
	A	Bean26a5	6.5 ± 1.1 (14)	RSc2508^A394(-)*^			1940	20
	C	Bean26c1	6.6 ± 1.1 (19)	*efpR* ^P93Q^	*purF* ^G-88A^		897	18
Cabbage var. Bartolo	B	Cab36b1	4.1 ± 0.4 (17)	RSp0955^IS, -1082^	*flhB* ^Dup 21 nt, 1129^		1494	14
	B	Cab36b2	4.9 ± 1.0 (12)	RSc2508^IS, 760^	RSp0955^IS, -1082^	*flhB* ^Dup 21 nt, 1129^	2038	18
	C	Cab36c2	8.8 ± 1.6 (13)	*spoT* ^A219P^	RSc2428^C-21A^	RSc2573-2622^Del 44.4kb^	1740	13
	D	Cab36d1	3.5 ± 0.4 (12)	*phcS* ^Y106C^	*flgB* ^Del 12 nt,483^	RSc2573-2622^Del 44.4kb^	2309	18
	E	Cab36e3	9.4 ± 1.1 (11)	RSc2573-2622^Del 44.4kb^			1515	12
Tomato var. Hawaii	A	Haw35a1	8.6 ± 1.5 (21)	*soxA1* ^C639R^			1227	14
	A	Haw35a4	7.2 ± 2.2 (20)				187	12
	B	Haw35b1	6.5 ± 1.0 (23)	RSp1574^V95L^			478	15
	B	Haw35b4	12.9 ± 3.3 (25)	RSp1574^V95L^	*prhP* ^IS, -6^		503	15
	C	Haw35c1	4.2 ± 1.6 (24)				902	21
	C	Haw35c2	4.0 ± 1.1 (24)				272	14
	D	Haw35d3	5.4 ± 1.3 (29)				125	14
	D	Haw35d5	4.1 ± 1.1 (27)				269	14
	E	Haw35e1	3.8 ± 1.0 (27)	RSp1136^C-218A^			245	16
	E	Haw35e3	5.4 ± 1.3 (24)	RSp1136^C-218A^	RSc3094^R162R^		212	14

Note: The CI value with the SE and the number of replicates is indicated for each evolved clone and was measured in planta in competition with the ancestral GMI1000 clone in our previous works [37,39]. In the Mutation column, the gene ID or gene name and the modification type is indicated. For SNPs inside the coding sequence, the protein modification is indicated with the original amino acid followed by the position of the SNP and by the new amino acid. For SNPs upstream the start codon of a gene, the original nucleotide is indicated followed by the position of the SNP from the start codon and by the new nucleotide. For small insertion (In), deletion (Del), and duplication (Dup), the size of the modification is indicated followed by the position of the modification. For IS insertion (IS), the position of the insertion is indicated upstream the start codon or in the coding sequence of the gene.

*Single nucleotide deletion; ns, not significantly different from the ancestral clone; nt, nucleotides.

The number of DEGs in each evolved clone was determined in our previous work from bacterial cultures in synthetic medium supplemented with glutamine and collected at the beginning of stationary phase (optical density around 1) [42]. The number of DMSs in each evolved clone was determined in the present work in the same culture conditions.

CI, competitive index; DEG, differentially expressed gene; DMS, differential methylated site; SE, standard error.

SMRT-seq data from the 31 evolved clones were investigated for methylome analysis in the same conditions as for the ancestral clone. Comparison of the methylation marks on the adenine of the GTWWAC motifs between the ancestral clone and the 31 evolved clones revealed a list of 50 DMSs. This list included 30 DMSs at 1 DNA strand (hemimethylated region) and 10 DMSs at both DNA strands (Tables 3, 4, and S1). Between 12 and 21 (15.5 ± 2.2; mean ± standard deviation) DMSs were detected per evolved clone (Tables 2, 3, and 4 and S1 Fig). The experimental host did not have a strong impact on the number of DMSs, with the exception that the number of DMSs detected in bean clones was significantly superior to the number of DMSs detected in eggplant Zebrina and in tomato Hawaii clones (S1 Fig). The number of mutations in each of the clones also had no impact on the number of detected DMSs (S2 Fig).

**Table 3:** DMSs on the chromosome between the ancestral clone and the clones evolved on 5 different plant species.

Gene ID	Motif	upstream / inside the ORF	*Gene name*	Gene function	Position (bp)	Ancestral clone	Clones evolved on Tomato var. Marmande						Clones evolved on Eggplant var. Zebrina							Clones evolved on Bean var. BP			Clones evolved on Cabbage var. Bartolo					Clones evolved on Tomato var. Hawaii 7996									
						GMI1000 methylation profile	a1	a2	b2	d2	e1	e3	b1	b5	c2	c3	c4	d1	e1	a4	a5	c1	b1	b2	c2	d1	e3	a1	a4	b1	b4	c1	c2	d3	d5	e1	e3
RSc0081	GTTAAC	upstream	* *	Transcriptional regulator, MurR/RpiR family	94117	6mA	6mA	6mA	6mA	6mA	6A	6mA	6mA	6mA	6mA	6mA	6mA	6mA	6A	6mA	6mA	6mA	6mA	6mA	6mA	6A	6mA	6mA	6mA	6mA	6mA	6A	6mA	6mA	6A	6mA	6mA
RSc0081	GTTAAC	upstream	* *	Transcriptional regulator, MurR/RpiR family	94120	6A	6A	6mA	6mA	6mA	6mA	6mA	6mA	6mA	6mA	6mA	6mA	6mA	6A	6A	6A	6mA	6mA	6mA	6mA	6A	6mA	6mA	6mA	6mA	6mA	6mA	6A	6mA	6A	6mA	6mA
RSc0102 / RSc0103	GTTAAC	upstream	*/ tISRso5*	Pseudogene: Ca2+-binding protein, RTX toxin-related (C-terminal fragment) / Transposase (ISRSO5 family)	117936	**6mA**	6mA	6mA	6mA	6mA	6mA	6mA	6mA	6mA	6mA	6mA	6mA	6mA	6mA	6mA	6mA	6mA	6mA	6mA	6mA	6mA	6mA	6mA	6mA	6mA	6mA	**6A**	6mA	6mA	6A	6mA	6mA
RSc0102 / RSc0103	GTTAAC	upstream	*/ tISRso5*	Pseudogene: Ca2+-binding protein, RTX toxin-related (C-terminal fragment) / Transposase (ISRSO5 family)	117939	**6mA**	6mA	6mA	6mA	6mA	6mA	6mA	6mA	6mA	6mA	6mA	6mA	6mA	6mA	6mA	6mA	6mA	6mA	6mA	6mA	6mA	6mA	6mA	6mA	6mA	6mA	**6A**	6mA	6mA	6mA	6mA	6mA
RSc0102 / RSc0103	GTAAAC	upstream	*/ tISRso5*	Pseudogene: Ca2+-binding protein, RTX toxin-related (C-terminal fragment) / Transposase (ISRSO5 family)	117950	6mA	6mA	6mA	6mA	6mA	6mA	6mA	6mA	6mA	6mA	6mA	6mA	6mA	6mA	6A	6mA	6mA	6mA	6mA	6mA	6mA	6mA	6mA	6mA	6mA	6mA	6mA	6mA	6mA	6mA	6mA	6mA
RSc0109 / RSc0110	GTTAAC	upstream	*thiG / tISRso5*	Thiazole synthase ThiG / Transposase (ISRSO5 family)	127847	6mA	6mA	6mA	6mA	6mA	6mA	6mA	6mA	6mA	6mA	6mA	6mA	6A	6mA	6mA	6mA	6mA	6mA	6mA	6mA	6mA	6mA	6mA	6mA	6mA	6mA	6mA	6mA	6mA	6mA	6mA	6mA
RSc0608	GTTAAC	upstream	*ripAA*	Type III effector protein RipAA	655714	6mA	6mA	6mA	6mA	6mA	6mA	6mA	6mA	6A	6mA	6mA	6mA	6mA	6mA	6mA	6mA	6mA	6mA	6mA	6A	6mA	6mA	6mA	6mA	6mA	6mA	6mA	6mA	6mA	6A	6A	6mA
RSc0608	GTTAAC	upstream	*ripAA*	Type III effector protein RipAA	655717	6A	6mA	6mA	6mA	6mA	6mA	6mA	6mA	6mA	6mA	6mA	6mA	6mA	6mA	6mA	6mA	6mA	6mA	6mA	6mA	6mA	6mA	6mA	6mA	6mA	6mA	6mA	6mA	6mA	6A	6mA	6mA
RSc0637	GTTAAC	upstream	*tISRso5*	ISRSO5-transposase protein	683376	6mA	6mA	6mA	6mA	6mA	6mA	6mA	6mA	6mA	6mA	6mA	6mA	6mA	6mA	6mA	6mA	6mA	6mA	6mA	6mA	6mA	6mA	6mA	6mA	6mA	6mA	6A	6mA	6mA	6A	6mA	6mA
RSc0637	GTTAAC	upstream	*tISRso5*	Transposase (ISRSO5 family)	683379	6mA	6mA	6mA	6mA	6mA	6mA	6mA	6mA	6mA	6mA	6mA	6mA	6mA	6mA	6mA	6mA	6mA	6mA	6mA	6mA	6mA	6mA	6mA	6mA	6mA	6mA	6mA	6mA	6A	6mA	6mA	6mA
RSc0958	GTTAAC	upstream	* *	Type VI secretion system tip VgrG family protein	1004576	**6A**	6A	6A	6A	6A	6A	6A	6A	6A	6A	6A	6A	6A	6A	6A	6A	6A	6A	**6mA**	6A	6mA	6A	6A	6A	6A	6A	6A	6A	6mA	6A	6A	6A
RSc0958	GTTAAC	upstream	* *	Type VI secretion system tip VgrG family protein	1004579	**6A**	6A	6A	6A	6A	6mA	6A	6A	6A	6mA	6mA	6mA	6mA	6A	6A	6mA	6A	6A	**6mA**	6mA	6A	6A	6A	6A	6A	6A	6A	6mA	6A	6A	6mA	6A
RSc1078 / RSc1079	GTAAAC	upstream	*/gudD1*	Transcription regulator / D-Glucarate dehydratase	1134729	6mA	6mA	6mA	6mA	6mA	6mA	6mA	6mA	6mA	6mA	6mA	6mA	6mA	6mA	6mA	6A	6mA	6mA	6mA	6mA	6mA	6mA	6mA	6mA	6mA	6mA	6mA	6mA	6mA	6mA	6mA	6mA
RSc1539	GTATAC	upstream	*sixA*	Phosphohistidine phosphatase SixA	1645829	6mA	6mA	6mA	6mA	6mA	6mA	6mA	6mA	6mA	6mA	6mA	6mA	6mA	6mA	6mA	6A	6mA	6mA	6mA	6mA	6mA	6mA	6mA	6mA	6mA	6mA	6mA	6mA	6mA	6mA	6mA	6mA
RSc2095	GTTAAC	upstream	*xdhA*	Xanthine Dehydrogenase, subunit A	2267250	6A	6mA	6mA	6mA	6mA	6mA	6mA	6mA	6mA	6mA	6mA	6mA	6mA	6mA	6mA	6mA	6mA	6mA	6mA	6mA	6mA	6mA	6mA	6mA	6mA	6mA	6mA	6mA	6mA	6mA	6mA	6mA
RSc2176	GTAAAC	upstream	*tISrso5*	ISRSO5-transposase protein	2360129	6A	6mA	6mA	6mA	6mA	6mA	6mA	6mA	6mA	6mA	6mA	6mA	6mA	6mA	6mA	6mA	6mA	6mA	6mA	6mA	6mA	6mA	6mA	6mA	6mA	6mA	6A	6mA	6A	6mA	6mA	6mA
RSc2176	GTAAAC	upstream	*tISrso5*	ISRSO5-transposase protein	2360143	6A	6mA	6mA	6mA	6mA	6mA	6mA	6mA	6mA	6mA	6mA	6mA	6mA	6mA	6mA	6mA	6mA	6mA	6mA	6mA	6mA	6mA	6mA	6mA	6mA	6mA	6mA	6mA	6mA	6mA	6mA	6mA
RSc2490 / RSc2492	GTTAAC	upstream	*icd/*	Isocitrate dehydrogenase / Acid phosphatase	2697793	6mA	6mA	6mA	6mA	6mA	6mA	6mA	6mA	6mA	6mA	6mA	6mA	6mA	6mA	6mA	6A	6mA	6mA	6mA	6mA	6mA	6mA	6mA	6mA	6mA	6mA	6mA	6mA	6mA	6mA	6mA	6mA
RSc2534	GTAAAC	upstream	* *	Oxidoreductase	2741386	6mA	6mA	6mA	6mA	6mA	6mA	6mA	6mA	6A	6mA	6mA	6mA	6mA	6mA	6mA	6mA	6mA	6mA	6mA	6mA	6mA	6mA	6mA	6mA	6mA	6mA	6mA	6mA	6mA	6mA	6mA	6mA
RSc2612	GTAAAC	intragenic	* *	ICE Tn4371 - Hypothetical protein	2813720	**6A**	6mA	6mA	6mA	6mA	6mA	6mA	6mA	6mA	6mA	6mA	6mA	6mA	6mA	6mA	6mA	6mA	6mA	6mA	na	na	na	6mA	6mA	6mA	6mA	6mA	6mA	6mA	6mA	6mA	6mA
RSc2612	GTAAAC	intragenic	* *	ICE Tn4371 - Hypothetical protein	2813723	**6A**	6mA	6mA	6mA	6mA	6mA	6mA	6mA	6mA	6mA	6mA	6mA	6mA	6mA	6mA	6mA	6mA	6mA	6mA	na	na	na	6A	6mA	6mA	6mA	6mA	6mA	6mA	6mA	6A	6mA
RSc2654	GTAAAC	upstream	* *	Peptidase, S8 family	2856885	6mA	6A	6mA	6mA	6mA	6A	6A	6mA	6mA	6mA	6mA	6mA	6mA	6A	6A	6A	6mA	6A	6mA	6mA	6A	6mA	6mA	6mA	6mA	6mA	6A	6mA	6mA	6A	6mA	6mA
RSc2918	GTAAAC	upstream	* *	RNA polymerase sigma factor, RpoE family protein	3145217	6mA	6mA	6mA	6mA	6mA	6mA	6mA	6mA	6mA	6mA	6mA	6mA	6mA	6A	6mA	6mA	6mA	6mA	6mA	6mA	6mA	6mA	6mA	6mA	6mA	6mA	6mA	6mA	6mA	6mA	6mA	6mA
RSc3177	GTTAAC	upstream	* *	Cyclic nucleotide binding domain-containing protein	3435906	6mA	6mA	6mA	6A	6A	6mA	6A	6mA	6mA	6mA	6mA	6mA	6mA	6A	6A	6mA	6A	6A	6mA	6A	6A	6mA	6mA	6mA	6A	6mA	6A	6A	6mA	6mA	6A	6mA
RSc3393	GTTAAC	upstream	*tISRso5*	Transposase (ISRSO5 family)	3660531	6mA	6mA	6mA	6mA	6mA	6mA	6mA	6mA	6A	6mA	6mA	6mA	6mA	6mA	6mA	6mA	6mA	6mA	6mA	6mA	6mA	6mA	6mA	6mA	6mA	6mA	6mA	6mA	6mA	6mA	6mA	6mA

Note: GTWWAC motifs were annotated intragenic if their positions mapped within the annotated coding sequence and upstream if they mapped to the first noncoding 300 bp before the annotated start codon. Newly methylated sites are indicated in blue and newly unmethylated sites in red. In grey boxes are indicated the 2 strand DMSs. The DMSs investigated by MSRE-qPCR are underlined and bold. BP, Blanc Précoce; bp, base pair. na, non-available data; in the Cab36c2, d1, and e3 clones, the RSc2612 gene is deleted (see Table 2).

**Table 4:** DMSs on the megaplasmid between the ancestral clone and the clones evolved on 5 different plant species.

Gene ID	Motif	upstream / inside the ORF	*Gene name*	Gene function	Position (bp)	Ancestral clone	Clones evolved on Tomato var. Marmande						Clones evolved on Eggplant var. Zebrina							Clones evolved on Bean var. BP			Clones evolved on Cabbage var. Bartolo					Clones evolved on Tomato var. Hawaii 7996									
						GMI1000 methylation profile	a1	a2	b2	d2	e1	e3	b1	b5	c2	c3	c4	d1	e1	a4	a5	c1	b1	b2	c2	d1	e3	a1	a4	b1	b4	c1	c2	d3	d5	e1	e3
RSp0077	GTTAAC	upstream	* *	Type II toxin-antitoxin system, RelE/ParE toxin family	87279	6mA	6mA	6mA	6mA	6mA	6mA	6mA	6mA	6mA	6mA	6mA	6mA	6mA	6mA	6mA	6mA	6mA	6mA	6mA	6mA	6mA	6mA	6mA	6mA	6mA	6A	6A	6mA	6mA	6mA	6mA	6mA
RSp0216 / RSp0217	GTTTAC	intragenic / upstream	*/tISRso5*	Pseudogene: Type 3 Secretion effector RipBM (C-terminal fragment) / ISRSO5-transposase protein	269725	6A	6mA	6mA	6mA	6mA	6mA	6mA	6mA	6mA	6mA	6mA	6mA	6mA	6mA	6mA	6mA	6mA	6mA	6mA	6mA	6mA	6mA	6mA	6mA	6mA	6mA	6mA	6mA	6mA	6mA	6mA	6mA
RSp0338	GTTTAC	upstream	*epsR*	Transcription Regulator EpsR	445723	**6A**	6A	6A	**6mA**	6A	6A	6A	6A	6A	6A	6A	6A	6A	6A	6A	6A	**6mA**	6A	6A	6A	**6mA**	6A	6A	6A	6A	6A	6A	6A	6A	6A	6A	6A
RSp0338	GTAAAC	upstream	*epsR*	Transcription Regulator EpsR	445726	**6A**	6A	6A	**6mA**	6A	6A	6A	6A	6A	6A	6A	6A	6A	6A	6A	6A	**6mA**	6A	6A	6A	**6mA**	6A	6A	6A	6A	6A	6A	6A	6A	6A	6A	6A
RSp0338	GTAAAC	upstream	*epsR*	Transcription Regulator EpsR	445735	**6A**	6A	6A	**6mA**	6A	6A	6A	6A	6A	6A	6A	6A	6A	6A	6A	6A	**6A**	6A	6A	6A	**6mA**	6A	6A	6A	6A	6A	6A	6A	6A	6A	6A	6A
RSp0338	GTTTAC	upstream	*epsR*	Transcription Regulator EpsR	445738	**6A**	6A	6A	**6mA**	6A	6A	6A	6A	6A	6A	6A	6A	6A	6A	6A	6A	**6mA**	6A	6A	6A	**6mA**	6A	6A	6A	6A	6A	6A	6A	6A	6A	6A	6A
RSp0449	GTAAAC	intragenic	* *	Pseudogene: RSH repeat protein (C-terminal fragment)	561449	6mA	6mA	6mA	6mA	6mA	6mA	6mA	6mA	6mA	6mA	6mA	6mA	6mA	6mA	6mA	6A	6mA	6mA	6mA	6mA	6mA	6mA	6mA	6mA	6mA	6mA	6mA	6mA	6mA	6mA	6mA	6mA
RSp0454	GTTAAC	intragenic	* *	Pseudogene: RHS repeat protein (N-terminal fragment)	565511	6mA	6mA	6mA	6mA	6mA	6mA	6mA	6mA	6mA	6mA	6mA	6mA	6mA	6mA	6mA	6mA	6mA	6mA	6mA	6mA	6mA	6mA	6mA	6mA	6mA	6mA	6mA	6mA	6mA	6mA	6A	6mA
RSp0629	GTTAAC	upstream	* *	Type 6 secretion system tip VgrG family protein with DUF2345 domain	765405	**6A**	6A	6mA	6A	6A	6A	6A	6A	6A	6A	6A	6A	6A	6A	6A	6A	6A	6A	**6mA**	6A	6A	6A	6A	6A	6A	6A	6A	6A	6A	6A	6A	6A
RSp0629	GTTAAC	upstream	* *	Type 6 secretion system tip VgrG family protein with DUF2345 domain	765408	**6A**	6A	6A	6A	6A	6A	6A	6A	6A	6A	6A	6A	6A	6A	6A	6A	6A	6A	**6mA**	6A	6A	6A	6mA	6A	6A	6A	6A	6A	6A	6A	6A	6A
RSp0641	GTAAAC	intragenic	*rmyB*	Ralsolamycin synthase, unit B	792207	6mA	6mA	6mA	6mA	6mA	6mA	6mA	6mA	6mA	6mA	6mA	6mA	6mA	6A	6mA	6mA	6mA	6mA	6mA	6mA	6mA	6mA	6mA	6mA	6mA	6mA	6mA	6mA	6mA	6mA	6mA	6mA
RSp0726	GTAAAC	intragenic	* *	Major facilitator superfamily (MFS) transporter	913370	6mA	6mA	6mA	6mA	6mA	6mA	6mA	6mA	6mA	6mA	6mA	6mA	6mA	6mA	6mA	6A	6mA	6mA	6mA	6mA	6mA	6mA	6mA	6mA	6mA	6mA	6mA	6mA	6mA	6mA	6mA	6mA
RS06160	GTAAAC	intragenic	* *	Ribosomal RNA-23S	1202810	6mA	6mA	6mA	6mA	6mA	6mA	6mA	6mA	6mA	6mA	6mA	6mA	6mA	6mA	6A	6mA	6mA	6mA	6mA	6mA	6mA	6mA	6mA	6mA	6mA	6mA	6mA	6mA	6mA	6mA	6mA	6mA
RSp1025	GTTTAC	upstream	* *	Translocator, LysE family	1298049	6A	6mA	6mA	6mA	6mA	6mA	6mA	6mA	6mA	6mA	6mA	6mA	6mA	6A	6mA	6mA	6mA	6mA	6mA	6A	6mA	6mA	6mA	6mA	6mA	6mA	6mA	6mA	6mA	6mA	6mA	6mA
RSp1152	GTTAAC	upstream	*tISRso5*	Transposase (ISRSO5 family)	1452556	**6mA**	6mA	6mA	6mA	6mA	6mA	6mA	6mA	6mA	6mA	6mA	6mA	6A	6mA	6mA	6mA	6mA	6mA	6mA	6mA	6mA	6mA	6mA	6mA	6mA	6mA	**6A**	6mA	6mA	6mA	6mA	6mA
RSp1152	GTTAAC	upstream	*tISRso5*	Transposase (ISRSO5 family)	1452559	**6mA**	6mA	6mA	6mA	6mA	6mA	6mA	6mA	6mA	6mA	6mA	6mA	6mA	6mA	6mA	6mA	6mA	6mA	6mA	6mA	6mA	6mA	6mA	6mA	6mA	6mA	**6A**	6mA	6mA	6mA	6mA	6mA
RSp1329	GTATAC	intragenic	* *	hypothetical protein	1680220	**6A**	6mA	6A	6mA	6mA	6mA	6mA	6mA	6mA	6mA	6mA	6mA	6mA	6mA	6mA	6mA	6mA	6mA	6mA	6mA	6mA	6mA	6mA	6A	6mA	6A	6A	6mA	6mA	6A	6mA	6mA
RSp1329	GTATAC	intragenic	* *	hypothetical protein	1680223	**6A**	6mA	6mA	6mA	6mA	6mA	6mA	6mA	6mA	6mA	6mA	6mA	6mA	6mA	6mA	6mA	6mA	6A	6mA	6A	6mA	6mA	6mA	6A	6mA	6A	6A	6A	6A	6A	6mA	6mA
RSp1529	GTAAAC	upstream	*efe*	1-aminocyclopropane-1-carboxylate oxidase (Ethylene-forming enzyme)	1916009	**6A**	6mA	6mA	6mA	6mA	6mA	6mA	6mA	6A	6mA	6mA	6mA	6A	6mA	6mA	6mA	6mA	6mA	6mA	6mA	6A	6mA	6mA	6mA	6mA	6mA	6mA	6mA	6mA	6A	6mA	6mA
RSp1529	GTAAAC	upstream	*efe*	1-aminocyclopropane-1-carboxylate oxidase (Ethylene-forming enzyme)	1916012	**6A**	6A	6A	6A	6mA	6A	6A	6A	6A	6A	6A	6A	6A	6A	6A	6A	6A	6A	6A	6A	6A	6mA	6A	6A	6A	6A	6A	6A	6A	6A	6A	6A
RSp1544	GTTAAC	intragenic	* *	hypothetical protein	1939052	6A	6mA	6A	6mA	6mA	6A	6mA	6mA	6mA	6mA	6mA	6A	6mA	6mA	6mA	6mA	6A	6A	6mA	6mA	6mA	6A	6mA	6mA	6mA	6mA	6mA	6mA	6mA	6A	6A	6mA
RSp1545	GTAAAC	intragenic	* *	Filamentous hemagglutinin family protein	1939296	6mA	6mA	6mA	6mA	6mA	6mA	6mA	6mA	6mA	6mA	6mA	6mA	6mA	6mA	6mA	6mA	6mA	6mA	6mA	6mA	6mA	6mA	6mA	6mA	6mA	6mA	6A	6mA	6mA	6mA	6mA	6mA
RSp1643	GTTAAC	upstream	* *	Hypothetical protein	2062924	**6mA**	6mA	6mA	6mA	6mA	6mA	6mA	6mA	6mA	6mA	6mA	6mA	6mA	6mA	6mA	6mA	6A	6mA	6mA	6mA	6mA	6mA	6mA	6mA	6mA	**6A**	6mA	6mA	6mA	6mA	6mA	6mA
RSp1643	GTTAAC	upstream	* *	Hypothetical protein	2062927	**6mA**	6mA	6mA	6mA	6mA	6mA	6mA	6mA	6mA	6mA	6mA	6mA	6mA	6mA	6mA	6mA	6mA	6mA	6mA	6mA	6mA	6mA	6mA	6mA	6mA	**6A**	6mA	6mA	6mA	6A	6mA	6mA
RSp1675	GTTAAC	upstream	*tISRso5*	ISRSO5-transposase protein	2087332	6A	6mA	6mA	6mA	6mA	6mA	6mA	6mA	6mA	6mA	6mA	6mA	6mA	6mA	6mA	6mA	6mA	6mA	6mA	6mA	6mA	6mA	6mA	6mA	6mA	6mA	6mA	6mA	6mA	6mA	6mA	6mA

Note: GTWWAC motifs were annotated intragenic if their positions mapped within the annotated coding sequence, upstream if they mapped to the first noncoding 300 bp before the annotated start codon. Newly methylated sites are indicated in blue and newly unmethylated sites in red. In grey boxes are indicated the 2 strand DMSs. In grey boxes are indicated the 2 strand DMSs. The DMSs investigated by MSRE-qPCR are underlined and bold. BP, Blanc Précoce; bp, base pair; eps, exopolysaccharides.

**Fig 2:**
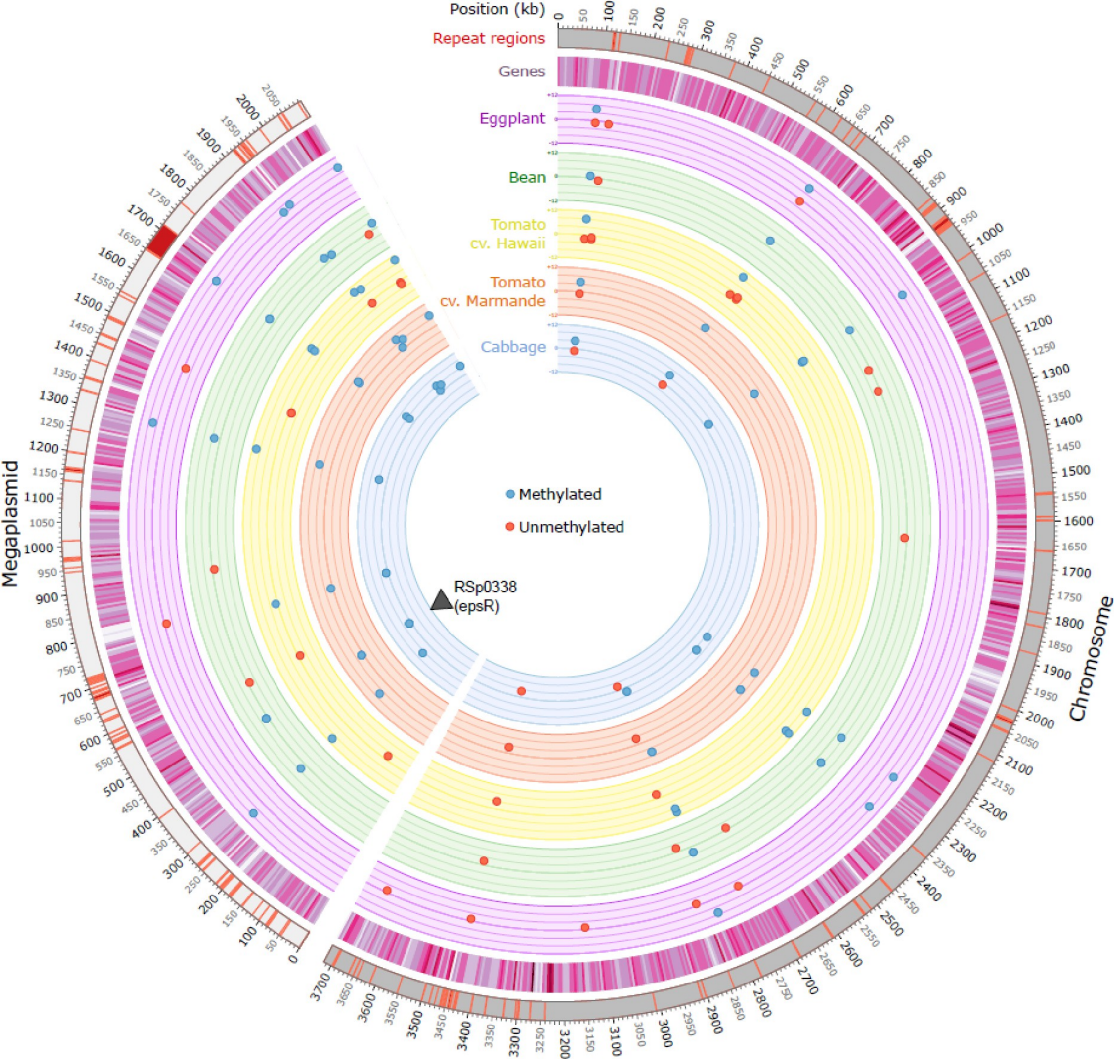
Circos plot highlighting the genomic repartition of the sites differentially methylated (DMS) between an ancestral clone and 31 clones evolved during 300 generations on 5 different plant species. Newly methylated sites are indicated in blue and unmethylated sites in red. A total of 31 evolved clones were investigated; 6 evolved on tomato cv. Marmande, 7 on eggplant, 3 on bean, 5 on cabbage, and 10 on tomato cv. Hawaii. The number of clones targeted by a DMS is indicated on the scale varying between 0 and 12 for each plant species. The black triangle indicates the position of the RSp0338 gene.

Genomic repartition analysis of the DMSs revealed that 26 were on the chromosome (3.7 Mb) and 24 on the megaplasmid (2.1 Mb), which seems to indicate a higher frequency on the second replicon (Table 5 and Fig 2). However, the examination of the map does not reveal any specific region enriched in newly methylated sites or unmethylated sites (Fig 2). DMSs can be classified as intragenic (position within a coding sequence), or intergenic either at the 5′ (upstream) or 3′ (downstream) position of a gene. Due to the existence of divergent promoters, a DMS at the 5′ position can potentially affect 2 genes, which explains why the number of genes potentially affected by these DMSs (39 genes) is slightly different from the number of DMSs (Table 5). Clearly, the number of DMSs positioned in a gene promoter region (defined as less than 300 nucleotides from the start codon) of the 39 affected genes is predominant (78%). Interestingly, 1 regulatory gene (RSp0338) has 2 GTWWAC motifs in its promoter region, both differentially methylated on both DNA strands (Table 4). An examination of the list of the DMSs affecting promoter regions revealed an overabundance of genes encoding TEs (33%). Of note, 14% of the GMI1000 TE sequences feature 1 or 2 GTWWAC motifs and 53% of these were targeted by a differential methylation mark. The examination of the list of the DMSs affecting promoter regions also revealed an overabundance of genes closely or remotely associated with virulence (RSp0338 encoding the *epsR* gene, RSp1529 encoding the *efe* gene, RSc0608 encoding the type III effector *ripAA*, and RSc0958 and Rsp0629 encoding VGR-related proteins linked to the type VI secretion system) (31%) (Table 5).

**Table 5:** General features of the DMSs identified in 31 clones evolved in 5 different plant species.

	Genome (5.8 Mbp)	Chromosome (3.7 Mbp)	Megaplasmid (2.1 Mbp)
**no. of DMSs**	**50**	**26**	**24**
*DMS frequency / Mbp*	*8*.*62*	*7*.*03*	*11*.*43*
**no. of genes affected by DMSs**	**39**	**22**	**17**
no. of DMSs in gene promoter regions*	**39**	**23**	**16**
*% DMSs in gene promoter regions*	*78*	*88*	*67*
**no. of DMSs affecting TEs**	**13**	**9**	**4**
*% TEs/no*. *of DMSs in gene promoter regions*	*33*	*39*	*25*
**no. of DMSs affecting virulence determinants**	**12**	**4**	**8**
*% virulence determinants/no*. *of DMSs in gene promoter regions*	*31*	*17*	*50*

*Gene promoter region was defined as the first noncoding 300 bp region before the annotated start codon of the gene (“upstream” region in Tables 3 and 4).

DMS, differential methylated site; Mbp, millions of base pairs; TE, transposable element.

### Differential methylation does not appear to be correlated with differential expression of the corresponding gene

**Fig 3:**
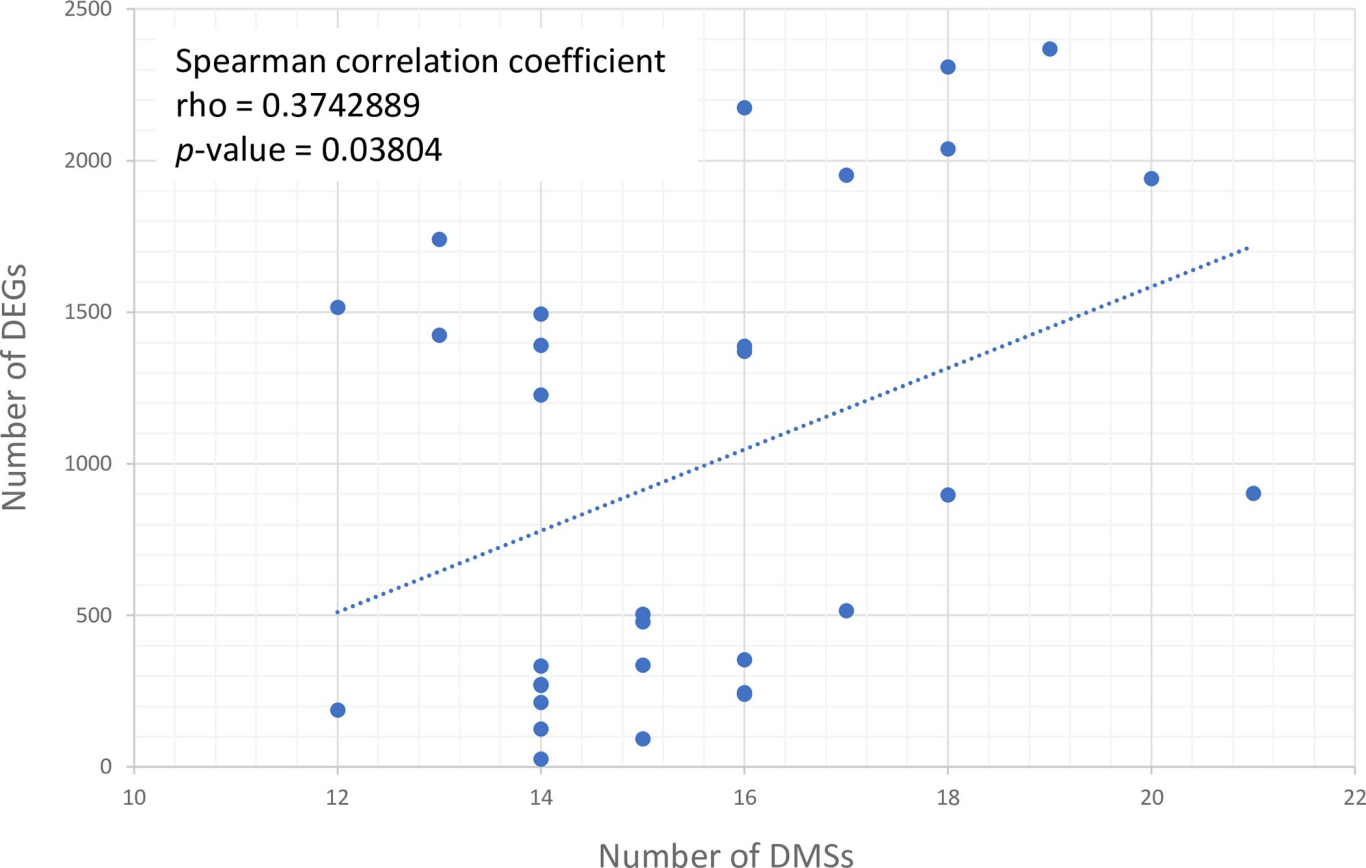
Correlation between the number of DMSs and DEGs in the 31 evolved clones. DEGs were identified in our previous work by sequencing of RNAs extracted from the same bacterial cultures used for methylome analysis (in synthetic medium supplemented with glutamine and collected at the beginning of stationary phase) [42]. DEGs between the evolved clones and the ancestral clone were considered as those presenting a log-fold change of expression I logFC I > 1 and an FDR-adjusted *p*-value (padj,FDR) < 0.05. Spearman’s rank correlation coefficient (rho) was calculated using the cor.test function from the stats R package and the *p*-value is indicated. The data underlying this figure can be found in S1 Data. DEG, differentially expressed gene; DMS, differential methylated site; FDR, false discovery rate.

Transcriptome analyses for the ancestral clone and the 31 evolved clones were performed in our previous work by sequencing of RNAs extracted from the same bacterial culture samples as those used for DNA extraction and methylome analysis (in synthetic MP medium and collected at the beginning of stationary phase) [42]. The number of differentially expressed genes (DEGs) for each clone was reported in Table 2. First, we estimated the correlation between the number of DMSs and the number of DEGs in each evolved clone. An analysis of the Spearman correlation coefficient showed a significant positive correlation between these 2 variables (*p*-value = 0.038), suggesting an impact of the number of differential methylation marks on the number of deregulated genes (Fig 3). In a second step, considering that gene expression is a population-based average, we wanted to estimate if a correlation could exist between methylation fraction and expression levels of a specific gene in the ancestral and the 31 evolved clones. However, the analysis of the Spearman correlation coefficient between the mean methylation fraction and the mean RNAseq counts for each gene in each clone did not find any correlation (S2 Table).

**Fig 4:**
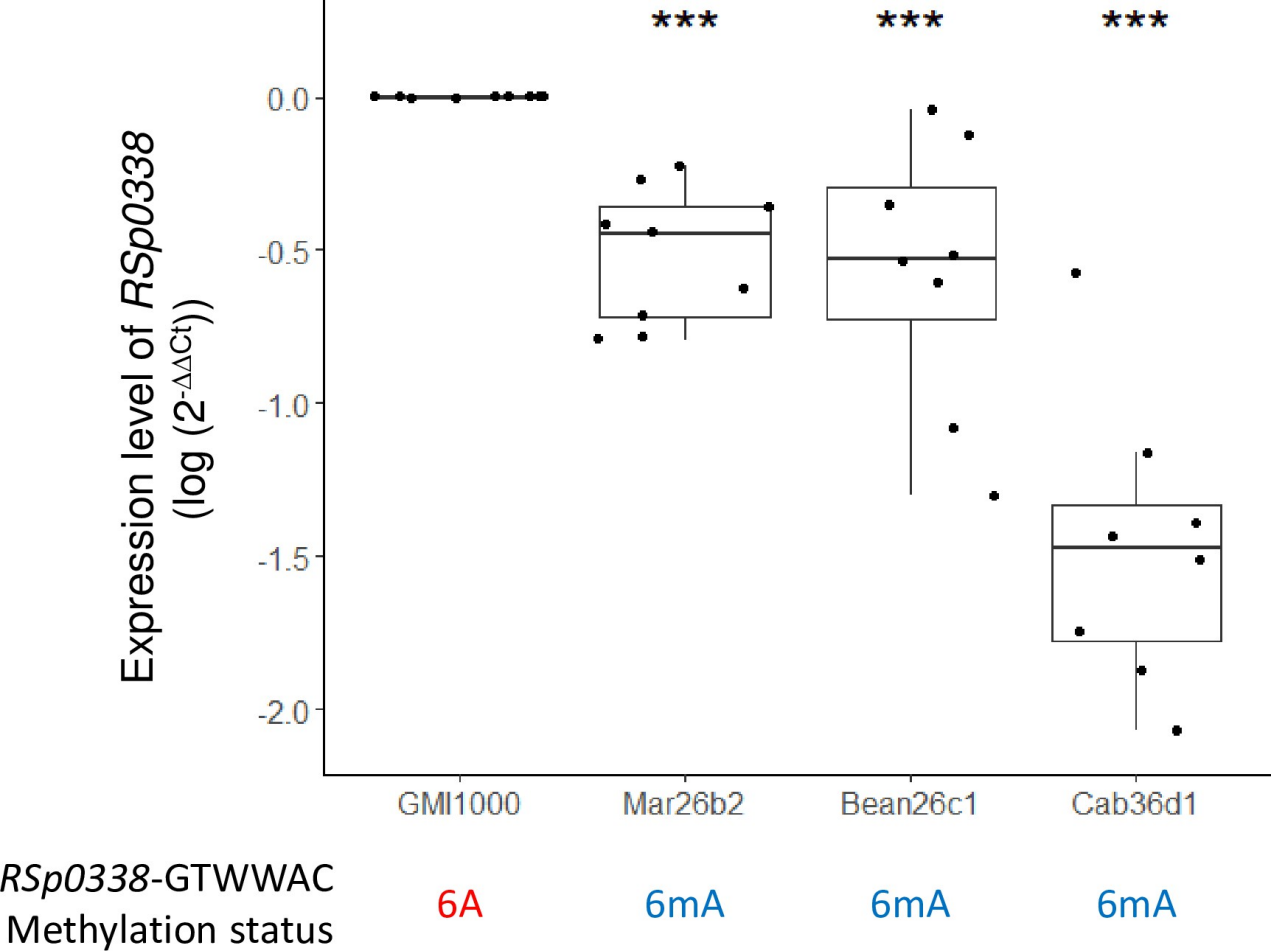
Relative expression level of RSp0338 gene between GMI1000 and evolved clones. Expression level of RSp0338 was determined during growth in synthetic medium supplemented with glutamine at the beginning of stationary phase, using an RT-qPCR approach. The methylation profile of the GTWWAC motifs in the upstream region of RSp0338 is indicated for each investigated clone. Three technical and 3 biological replicates were performed. Data were normalized using the 2^–ΔΔCt^ calculation method [49] and compared using a Wilcoxon test, ** *p*-value < 0.01. The data underlying this figure can be found in S1 Data.

In a third step, we wondered if the differential methylation of a given gene could impact its own expression. For that purpose, we conducted a Fisher’s exact test to determine whether there was an association between differential methylation and differential expression of the corresponding gene. As no correlation could be found using the differential gene expression threshold of I logFC I > 1; *p*-value FDR value < 0.05 nor I logFC I > 0.5; *p*-value FDR < 0.05, we relaxed the threshold to I logFC I > 0.5; *p*-value < 0.05 and *p*-value FDR < 0.08. Table 6 gives a summary of the relative gene expression in the experimentally evolved clones compared to the ancestral clone for each of the 39 genes targeted by a DMS using this last threshold. This analysis revealed that most of the DMSs did not affect expression of the targeted gene. A significant correlation was found only for the RSp0338 gene (Table 7). Down-regulation of the RSp0338 gene in the Mar26b2, Bean26c1, and Cab36d1 clones, in which the DMS was detected, compared to the ancestral GMI1000 clone was investigated using a quantitative reverse transcription PCR (RT-qPCR) approach. This analysis confirmed that the RSp0338 gene is down-regulated in the 3 investigated evolved clones compared to the ancestral GMI1000 clone (Fig 4).

**Table 6:** Relative gene expression in the experimentally evolved clones compared to the ancestral clone for each gene targeted by a DMS.

Gene ID	Tomato var. Marmande						Eggplant var. Zebrina							Bean var. BP			Cabbage var. Bartolo					Tomato var. Hawaii 7996									
	a1	a2	b2	d2	e1	e3	b1	b5	c2	c3	c4	d1	e1	a4	a5	c1	b1	b2	c2	d1	e3	a1	a4	b1	b4	c1	c2	d3	d5	e1	e3
RSc0081	0.24	**0.03**	**-2.40**	**0.41**	**0.23**	**-0.26**	**-1.12**	**0.35**	**-0.23**	**0.81**	**0.25**	**-0.13**	**-0.40**	-2.25	-2.33	**-0.76**	**-0.39**	**-0.91**	**-1.09**	**-0.09**	**-0.58**	**-0.52**	**-0.49**	**0.51**	**-0.19**	**-0.24**	-0.47	**0.13**	**0.78**	**0.47**	**0.44**
RSc0102	4.64	4.71	5.88	3.95	3.12	4.37	0.34	-0.48	0.07	-0.55	-0.35	0.56	0.59	**0.43**	0.26	1.12	4.85	4.23	4.51	5.74	4.45	1.53	0.29	0.26	0.92	**0.86**	0.31	-0.21	**-0.59**	-0.31	-0.72
RSc0103	-0.69	-0.78	0.29	-1.98	-2.88	-1.75	0.22	0.81	0.44	0.22	0.15	1.09	-0.21	**-0.45**	-0.03	1.38	-0.95	-1.49	-1.19	-0.10	-1.33	0.53	-0.15	0.27	0.40	**0.87**	0.24	0.03	**0.06**	-0.41	-0.71
RSc0109	-0.29	-0.38	-2.07	0.54	0.97	0.05	-1.30	-0.24	-0.92	0.75	-0.85	**-1.08**	0.41	0.30	0.70	0.17	-0.19	-0.67	-1.48	-0.20	-0.21	0.14	-0.72	-0.11	-0.44	-0.07	-0.52	-0.26	0.21	0.23	-0.04
RSc0110	1.46	1.21	-0.38	1.48	0.32	1.55	0.57	0.64	0.54	-0.10	0.46	**0.36**	-0.52	-0.52	-0.26	0.66	1.62	1.33	1.59	1.60	1.92	0.42	0.56	0.72	0.56	1.61	1.56	1.41	1.26	1.92	1.63
RSc0608	**-0.06**	**0.30**	**0.40**	**0.84**	**-0.14**	**0.55**	**-0.29**	**0.32**	**-0.52**	**-0.43**	**-0.34**	**0.14**	**0.81**	**0.01**	**-0.39**	**-2.02**	**1.52**	**-0.01**	**-0.92**	**-1.47**	**0.73**	**-0.15**	**0.06**	**-1.75**	**-1.51**	**-2.79**	**-2.34**	**-0.81**	**-0.85**	**-1.02**	**-1.38**
RSc0637	-1.60	-1.30	-0.20	-1.87	-2.52	-1.75	-0.19	-0.53	0.48	-0.75	-0.88	0.29	-0.69	-0.60	-0.77	-0.53	-1.24	-1.35	-0.70	-0.39	-1.59	-0.56	-0.07	-0.52	-0.71	**-0.60**	-0.25	**-1.37**	**-0.88**	-1.26	-1.15
RSc0958	-0.27	-0.12	0.67	-0.36	**-0.32**	-0.10	0.24	0.04	**0.40**	**-0.18**	**0.14**	**0.43**	0.24	0.51	**0.51**	0.35	-0.05	**0.28**	**0.64**	**0.08**	0.04	0.22	0.44	-0.02	0.49	0.14	**0.25**	**0.04**	-0.21	**-0.32**	-0.17
RSc1078	3.31	2.85	0.76	3.61	3.03	2.91	0.10	0.51	-0.15	0.40	0.80	-0.33	0.65	0.49	**0.52**	-0.61	2.62	1.94	1.80	3.29	2.96	-0.14	-0.66	0.40	0.05	0.09	0.13	0.28	-0.11	0.26	0.81
RSc1079	0.05	-0.21	-1.31	0.15	-0.98	0.22	-0.79	0.23	-0.41	0.38	-0.21	-0.39	0.40	-1.07	**-1.29**	-0.13	0.30	-0.01	-0.91	0.62	0.30	-0.09	-0.52	-0.13	-0.21	0.37	-0.08	0.06	0.21	-0.17	0.06
RSc1539	0.16	0.19	-0.39	0.55	-0.12	0.27	-0.23	0.37	-0.57	-0.99	-0.05	-1.48	0.63	-0.34	**-0.95**	-0.45	0.40	-0.36	-0.78	0.70	-0.40	0.05	0.03	-0.06	0.04	-0.02	0.12	-0.41	0.30	0.50	0.52
RSc2095	**0.89**	**0.40**	**-0.10**	**0.27**	**0.63**	**0.41**	**0.43**	**0.57**	**0.54**	**0.36**	**0.64**	**0.54**	**0.27**	**0.82**	**0.30**	**0.29**	**0.66**	**-0.31**	**0.57**	**1.43**	**0.41**	**0.90**	**0.45**	**0.73**	**0.59**	**1.30**	**0.67**	**0.62**	**0.58**	**0.62**	**0.55**
RSc2176	**-1.82**	**-1.40**	**-0.08**	**-2.26**	**-2.63**	**-1.72**	**0.10**	**-0.74**	**-0.26**	**-0.75**	**-0.64**	**-0.22**	**-1.29**	**-0.43**	**-0.30**	**-0.63**	**-2.31**	**-1.78**	**-0.53**	**-2.10**	**-1.49**	**-0.58**	**-0.08**	**-1.23**	**-1.00**	**-1.18**	**-0.88**	**-0.91**	**-1.00**	**-1.18**	**-1.13**
RSc2490	-0.60	-0.30	-0.05	-0.10	-0.19	0.12	0.13	0.28	0.57	0.01	0.46	0.56	-0.31	1.00	**0.80**	0.26	-0.03	0.85	-0.13	-0.63	-0.02	-0.03	-0.01	-0.49	-0.61	-0.32	-0.03	-0.07	-0.24	-0.22	-0.38
RSc2491	0.32	0.28	0.96	-0.70	-0.34	0.05	-0.03	0.14	-0.17	-0.03	-0.94	0.93	1.00	0.60	**0.03**	-0.04	0.56	1.23	1.01	-0.35	0.40	0.95	0.90	0.41	0.11	-0.18	0.87	1.09	0.81	0.76	0.79
RSc2492	0.26	-0.14	-1.30	-0.37	-0.63	-0.22	-0.33	-0.57	-0.40	0.04	-0.34	-0.45	0.22	-0.25	**-0.64**	-0.26	-0.53	-0.95	-0.34	-0.03	-0.10	-0.20	-0.31	-0.04	-0.19	-0.02	-0.29	-0.57	-0.15	-0.15	-0.30
RSc2534	1.23	0.66	-1.02	1.00	-0.24	-0.19	-1.53	**0.58**	-0.36	0.93	0.20	-0.09	1.09	-0.44	-0.32	-0.30	0.61	-1.12	-2.07	2.07	-0.33	0.78	-0.46	0.41	-0.91	1.26	-0.50	0.20	0.35	0.76	0.68
RSc2612	**-1.28**	**-0.75**	**0.78**	**-1.31**	**-0.70**	**-1.50**	**-0.35**	**-0.51**	**-0.16**	**-0.37**	**-0.37**	**0.59**	**-0.45**	**0.45**	**0.71**	**0.67**	**-0.60**	**0.87**	na	na	na	**-0.52**	**-0.10**	**-1.27**	**-0.71**	**-0.46**	**-0.17**	**-0.50**	**-0.65**	**-1.29**	**-1.14**
RSc2654	**-0.29**	-0.40	0.33	-0.36	**0.08**	**-0.38**	-0.12	-0.08	-0.30	-0.05	-0.27	-0.02	**-0.63**	**0.01**	**0.04**	-0.01	**-0.03**	0.02	0.09	**-0.29**	-0.46	-0.29	0.04	0.31	0.03	**-0.03**	0.24	0.02	**0.09**	-0.03	0.02
RSc2918	0.14	0.12	-1.15	-0.03	0.81	-0.16	0.08	-0.21	-0.32	-0.02	0.42	0.01	**-0.59**	1.19	1.58	0.15	-0.42	0.89	0.45	-0.90	-0.02	0.36	0.37	0.68	0.50	0.24	-0.01	0.48	0.51	0.42	0.31
RSc3177	1.36	1.22	**2.51**	**0.50**	-0.89	**0.73**	0.72	1.37	0.70	0.50	0.66	1.39	**1.03**	**-0.21**	0.01	**1.68**	**2.07**	0.37	**1.47**	**2.41**	0.87	2.19	1.22	**1.18**	1.63	**1.95**	**0.92**	1.07	0.80	**0.66**	0.66
RSc3393	4.82	5.41	5.86	3.93	2.54	4.40	-1.13	**-1.74**	-0.03	-0.81	-1.23	-0.22	-0.66	-1.21	-1.24	-0.68	4.47	4.57	4.50	5.43	4.91	0.02	0.11	-0.88	-0.81	0.01	-0.04	-0.59	-1.25	-1.44	-0.60
RSp0077	0.56	0.22	0.60	-0.67	-2.18	-0.06	-0.32	-0.07	0.18	-0.43	0.02	0.20	-0.13	-1.91	-1.09	0.42	0.33	-1.07	-0.13	1.42	0.15	0.51	0.10	0.05	**-0.19**	**0.78**	0.43	-0.01	0.11	-0.42	0.18
RSp0216	**0.14**	**0.40**	**2.19**	**-0.16**	**0.61**	**0.04**	**0.09**	**-0.26**	**0.41**	**-0.53**	**-0.16**	**0.50**	**0.21**	**1.07**	**0.57**	**0.55**	**0.51**	**0.98**	**0.95**	**0.92**	**0.71**	**0.94**	**0.36**	**0.01**	**0.12**	**0.57**	**0.28**	**-0.12**	**-0.45**	**-0.49**	**-0.40**
RSp0217	**-0.12**	**-0.15**	**0.97**	**-0.97**	**-0.54**	**-0.74**	**-0.04**	**-0.37**	**-0.01**	**-0.96**	**-0.39**	**-0.14**	**-1.19**	**-1.20**	**-1.10**	**-0.54**	**-0.37**	**-0.10**	**0.60**	**0.42**	**-0.60**	**-0.65**	**-0.25**	**-0.86**	**-0.84**	**-1.13**	**-0.80**	**-1.13**	**-0.74**	**-1.18**	**-1.15**
RSp0338	-0.56	0.001	**-1.27**	-1.03	-0.22	-0.67	0.04	-0.39	0.51	0.07	0.23	0.58	-0.48	0.33	0.40	**-0.83**	-0.71	0.70	0.09	**-2.89**	-0.36	-1.67	0.21	-1.10	-0.99	-1.28	0.02	-0.08	-0.08	-0.24	-0.51
RSp0449	-0.19	-0.15	1.67	-0.91	-1.95	-0.60	-0.06	-0.45	-0.14	-0.60	-0.51	0.37	0.19	-1.52	**-1.61**	0.16	0.29	-0.61	0.84	1.32	-0.07	0.33	0.59	-0.48	0.60	-0.03	-0.03	-0.25	-0.59	-0.75	-0.75
RSp0454	-0.73	-0.43	1.69	-1.10	-0.63	0.04	0.55	-1.07	-0.16	-1.33	-0.43	-0.87	0.08	0.51	0.03	0.21	-0.28	-0.18	0.59	0.25	0.33	0.98	0.57	-0.47	0.48	0.09	-0.38	-0.91	-1.13	**-0.92**	-1.08
RSp0629	0.62	**0.68**	1.28	0.43	1.18	0.66	0.50	-0.35	0.15	-0.26	-0.12	0.05	0.32	1.87	1.72	0.25	0.35	**1.41**	1.22	0.65	0.95	**0.54**	0.17	0.46	0.69	0.43	0.19	-0.11	-0.23	0.05	-0.14
RSp0641	0.36	0.42	0.87	1.24	0.98	1.75	0.79	1.36	0.54	0.20	0.38	0.15	**1.83**	1.39	1.32	1.50	1.48	0.94	0.22	-1.10	1.14	1.51	0.31	1.02	1.77	1.58	0.12	0.31	0.43	0.52	0.85
RSp0726	0.21	0.95	2.27	-0.17	-0.20	0.12	-0.01	-0.33	0.22	-0.48	-0.22	0.35	0.28	0.42	**0.53**	0.43	0.49	0.73	1.51	0.26	0.45	0.58	0.31	-0.03	0.31	0.42	0.22	-0.27	-0.34	-0.54	-0.42
RSp1025	**-1.35**	**-1.19**	**-0.68**	**-0.86**	**-0.68**	**-1.27**	**0.75**	**0.21**	**0.02**	**-0.20**	**-0.05**	**0.30**	-0.79	**-0.38**	**-0.48**	**-0.80**	**-1.38**	**-0.70**	-0.03	**-3.49**	**-1.29**	**-0.78**	**0.36**	**-0.98**	**-1.85**	**-1.75**	**-1.04**	**-0.31**	**-0.38**	**-0.25**	**-0.54**
RSp1152	-0.53	-0.47	0.15	-1.65	-2.47	-1.24	-0.48	0.12	0.27	0.02	0.17	**0.51**	0.13	-1.04	-0.82	0.38	-0.76	-1.79	-0.44	0.14	-1.72	0.61	0.02	0.06	0.23	**0.39**	-0.27	-0.38	-0.14	-0.73	-1.06
RSp1329	na	na	na	na	na	na	na	na	na	na	na	na	na	na	na	na	na	na	na	na	na	na	na	na	na	na	na	na	na	na	na
RSp1529	**1.04**	**1.11**	**1.75**	**0.44**	**-0.30**	**0.65**	**0.09**	-0.39	**0.05**	**-0.74**	**-0.44**	-0.15	**-0.31**	**-0.01**	**-0.10**	**-0.19**	**1.91**	**0.41**	**2.70**	0.95	**0.74**	**0.42**	**0.28**	**0.19**	**0.10**	**-0.03**	**-0.20**	**-0.30**	-0.89	**-0.40**	**-0.22**
RSp1544	**-1.64**	-1.38	**1.50**	**-2.35**	-2.48	**-2.06**	**-0.18**	**-0.18**	**-0.08**	**-0.31**	-0.41	**0.74**	**-0.53**	**-0.86**	**-0.90**	0.12	-0.92	**-1.18**	**0.27**	**-0.35**	-1.58	**-0.18**	**0.42**	**-0.92**	**0.27**	**-0.66**	**-0.53**	**-0.83**	-1.11	-1.65	**-1.48**
RSp1545	-0.12	-0.14	0.76	-0.69	-1.05	-0.53	-0.34	-0.70	-0.22	-0.57	-0.78	0.40	-0.40	-1.04	-1.07	-0.20	-0.19	-0.41	0.29	0.44	-0.24	-0.31	0.17	-0.55	0.13	**-0.35**	-0.50	-0.66	-0.81	-1.28	-1.02
RSp1643	1.00	1.46	1.63	0.63	0.60	1.23	0.44	-0.27	0.05	-0.45	-1.21	0.02	0.89	0.13	0.53	**-0.45**	1.39	1.56	1.51	1.36	1.39	-0.97	0.18	1.11	**1.87**	-0.79	-0.20	-0.29	**-0.57**	-0.67	-0.46
RSp1675	**0.44**	**0.63**	**1.54**	**-0.35**	**-0.93**	**0.10**	**-0.73**	**-1.04**	**0.22**	**-0.81**	**-0.63**	**0.30**	**-0.71**	**-1.99**	**-1.20**	**-0.32**	**0.60**	**-0.19**	**0.85**	**1.23**	**0.06**	**-0.05**	**-0.10**	**-0.70**	**-0.84**	**-1.00**	**-0.10**	**-0.96**	**-1.17**	**-1.38**	**-1.06**

Note: RNAseq analysis was conducted with RNAs extracted from the same bacterial cultures used for methylome analysis (in synthetic medium supplemented with glutamine and collected at the beginning of stationary phase). RNAseq raw data and analysis are given in [42]. The table gives the log Fold change values. In green are the down-regulated genes and in yellow the up-regulated genes (I logFC I > 0.5; *p*-value < 0.05; *p*-value FDR < 0.08). The values in bold and underlined indicate the clone in which the gene was targeted by a DMS (Tables 3 and 4). na, non-available data; in the Cab36c2, d1, and e3 clones, the RSc2612 gene is deleted (see Table 2).

**Table 7:** Association analysis between differential methylation and differential gene expression between the ancestral clone and the experimentally evolved clones.

Gene ID	DMS-DEG	DMS-non DEG	non DMS-DEG	non DMS-non DEG	*p-*value (Fisher exact test)
RSc0081	8	19	2	2	0.5773
RSc0102	2	1	15	13	1.000
RSc0103	1	2	10	18	1.000
RSc0109	1	0	6	24	0.2258
RSc0110	0	1	16	14	0.4839
RSc0608	12	19	0	0	1.000
RSc0637	1	2	12	16	1.000
RSc0958	2	10	2	17	0.6304
RSc1078	0	1	10	20	1.000
RSc1079	1	0	7	23	0.2581
RSc1539	0	1	0	30	1.000
RSc2095	13	18	0	0	1.000
RSc2176	20	11	0	0	1.000
RSc2490	1	0	5	25	0.1935
RSc2491	0	1	13	17	1.000
RSc2492	1	0	3	27	0.129
RSc2534	0	1	11	19	1.000
RSc2612	7	21	0	0	1.000
RSc2654	1	9	0	21	0.3226
RSc2918	0	1	4	26	1.000
RSc3177	11	2	12	6	0.412
RSc3393	0	1	12	18	1.000
RSp0077	1	1	5	24	0.3548
RSp0216	8	23	0	0	1.000
RSp0217	18	13	0	0	1.000
RSp0338	3	0	5	23	0.01246
RSp0449	1	0	11	19	0.3871
RSp0454	1	0	11	19	0.3871
RSp0629	3	0	10	18	0.06363
RSp0641	1	0	18	12	1.000
RSp0726	1	0	6	24	0.2258
RSp1025	15	14	0	2	0.4839
RSp1152	0	2	10	19	1.000
RSp1329	na	na	na	na	na
RSp1529	8	19	2	2	0.5773
RSp1544	12	11	6	2	0.412
RSp1545	0	1	15	15	1.000
RSp1643	1	2	11	17	1.000
RSp1675	11	20	0	0	1.000

Note: For each gene targeted by a DMS in at least 1 experimentally evolved clone, the table gives the number of clones in which the gene is both differentially methylated and differentially expressed (DMS-DEG (differentially expressed gene)), differentially methylated but not differentially expressed (DMS-non DEG), not differentially methylated but differentially expressed (non DMS-DEG) and neither differentially methylated nor expressed (non DMS—non DEG). A Fisher exact test was used to determine whether there was an association between methylation and gene expression. The grey boxes indicate the *p*-value < 0.05 and the corresponding gene.

### Assessment of methylation status through the MSRE-qPCR approach

We used MSRE-qPCR (methylation-sensitive restriction enzyme-quantitative PCR) assay as an alternative approach to assess the methylation status of DMSs identified by SMRT-seq. Briefly, MSRE-qPCR is based on extensive digestion of genomic DNA with methylation-sensitive restriction enzyme (MSRE) followed by quantitative PCR amplification of the target gene [50]. With this method, we could only test two-strand-DMSs, but not hemimethylated sites. Genomic DNA was prepared under the same conditions as for SMRT-seq.

**Fig 5:**
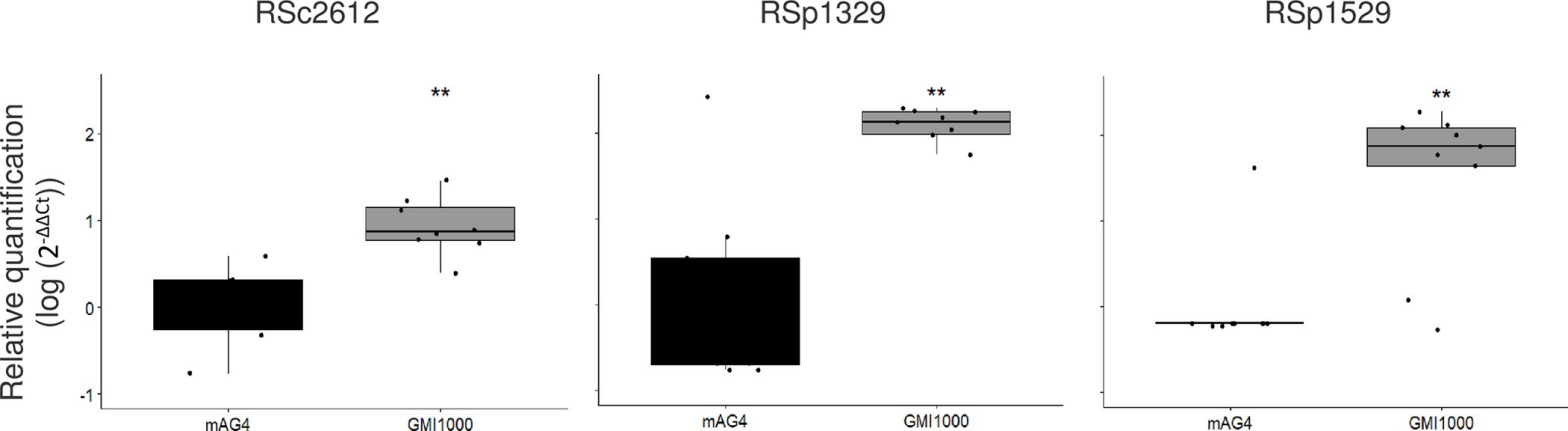
MSRE-qPCR results for analysis of methylation status of GTWWAC motifs at the RSc2612, RSp1329, and RSp1529 genes in the ancestral GMI1000 clone. The methylation profile of the GTWWAC motifs at the RSc2612, RSp1329, and RSp1529 genes for the ancestral GMI1000 clone was investigated using an MSRE-qPCR approach (methylation-sensitive restriction enzyme-quantitative PCR). Bacterial cells were grown in synthetic medium with glutamine, and DNA was recovered at the beginning of stationary phase. The mAG4 mutant (GMI1000 deleted from the RSc1982 MTase, targeting GTWWAC motifs) was used as a nonmethylated control at GTWWAC motifs. The graphs represent a relative quantification using the 2^−ΔΔCt^ method compared to the mAG4 mutant. Detection of an amplicon revealed that no digestion occurred and that the region was methylated, while no amplification revealed that the region was nonmethylated and digested. 2^−ΔΔCt^ values were compared between the ancestral clone and mAG4 mutant using a Wilcoxon test; ** *p*-value < 0.01. The data underlying this figure can be found in S1 Data.

The MSRE-qPCR approach was first used to assess the methylation status of the GMI1000 strain for 3 motifs that were detected methylated on both DNA strands for a majority of the evolved clones but not methylated in the ancestral clone according to SMRT-seq, thus suggesting a false-negative detection in the GMI1000 strain. These 3 motifs were associated with the RSc2612, RSp1329, and RSp1529 genes (Tables 3 and 4). The MSRE-qPCR results obtained for the ancestral clone were significantly different from the nonmethylated control mAG4, thus suggesting that the ancestral DNA was methylated at these 3 motifs such as the evolved DNA (Fig 5).

**Fig 6:**
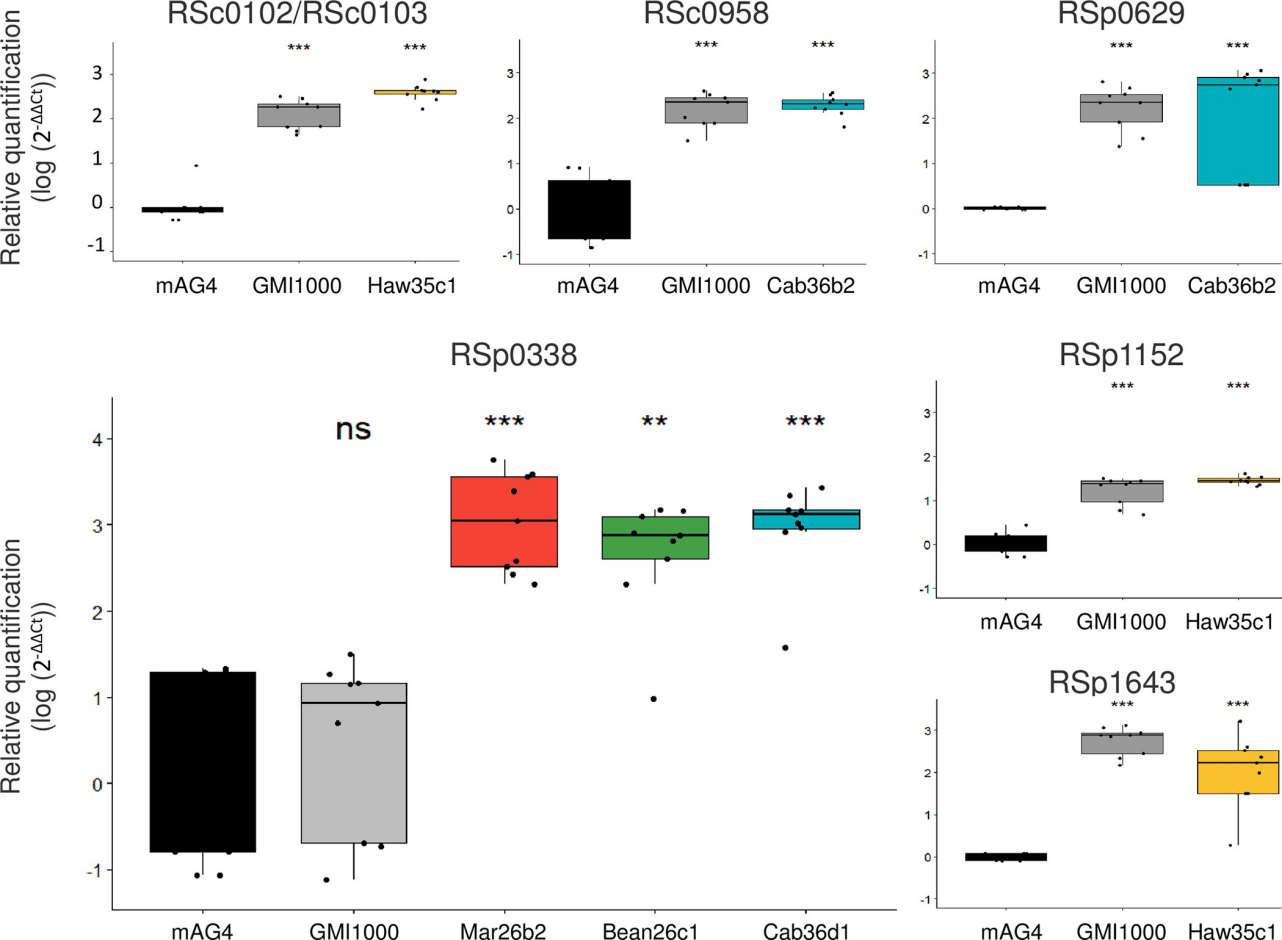
MSRE-qPCR results for analysis of methylation status of GTWWAC motifs upstream the RSc0102, RSc0958, RSp0338, RSp0629, RSp1152, and RSp1643 genes in the ancestral GMI1000 clone and the experimentally evolved clones. See Fig 5 for legend. 2^−ΔΔCt^ values were compared between the evolved or ancestral clone and mAG4 mutant using a Wilcoxon test; ns, not significant; * *p*-value < 0.05; ** *p*-value < 0.01; *** *p*-value < 0.001. The data underlying this figure can be found in S1 Data.

We then used MSRE-qPCR to investigate the methylation status of the 7 other motifs found differentially methylated on both DNA strands according to SMRT-seq. These 7 motifs included 1 motif in the divergent promoter region of both RSc0102 and RSc0103, 2 motifs upstream of RSp0338, and 1 motif upstream of RSc0958, RSp0629, RSp1152, and RSp1643 (Tables 3 and 4). MSRE-qPCR analysis was conducted on both GMI1000 DNA and DNA from the evolved clones in which the DMS was found (Tables 3 and 4). Again, most of the MSRE-qPCR results obtained for both the ancestral and evolved clones were significantly different from the nonmethylated control mAG4 (Fig 6). Thus, using this approach, the GTWWAC motifs upstream of RSc0102/RSc0103, RSc0958, RSp0629, RSp1152, and RSp1643 were not found differentially methylated between the ancestral and evolved clones, appearing fully methylated in both. Only the MSRE-qPCR results for the region upstream of RSp0338 revealed a differential methylated state between GMI1000 and the 3 independent clones evolved on tomato var. Marmande, bean, and cabbage. In agreement with SMRT-seq data, the GTWWAC motifs upstream of RSp0338 were not methylated in the ancestral clone but methylated in the Mar26b2, Bean26c1, and Cab36d1 evolved clones (Fig 6).

### Methylation upstream of the RSp0338 gene appeared after only 2 passages in tomato Marmande and remains stable after growth in a different medium

**Fig 7:**
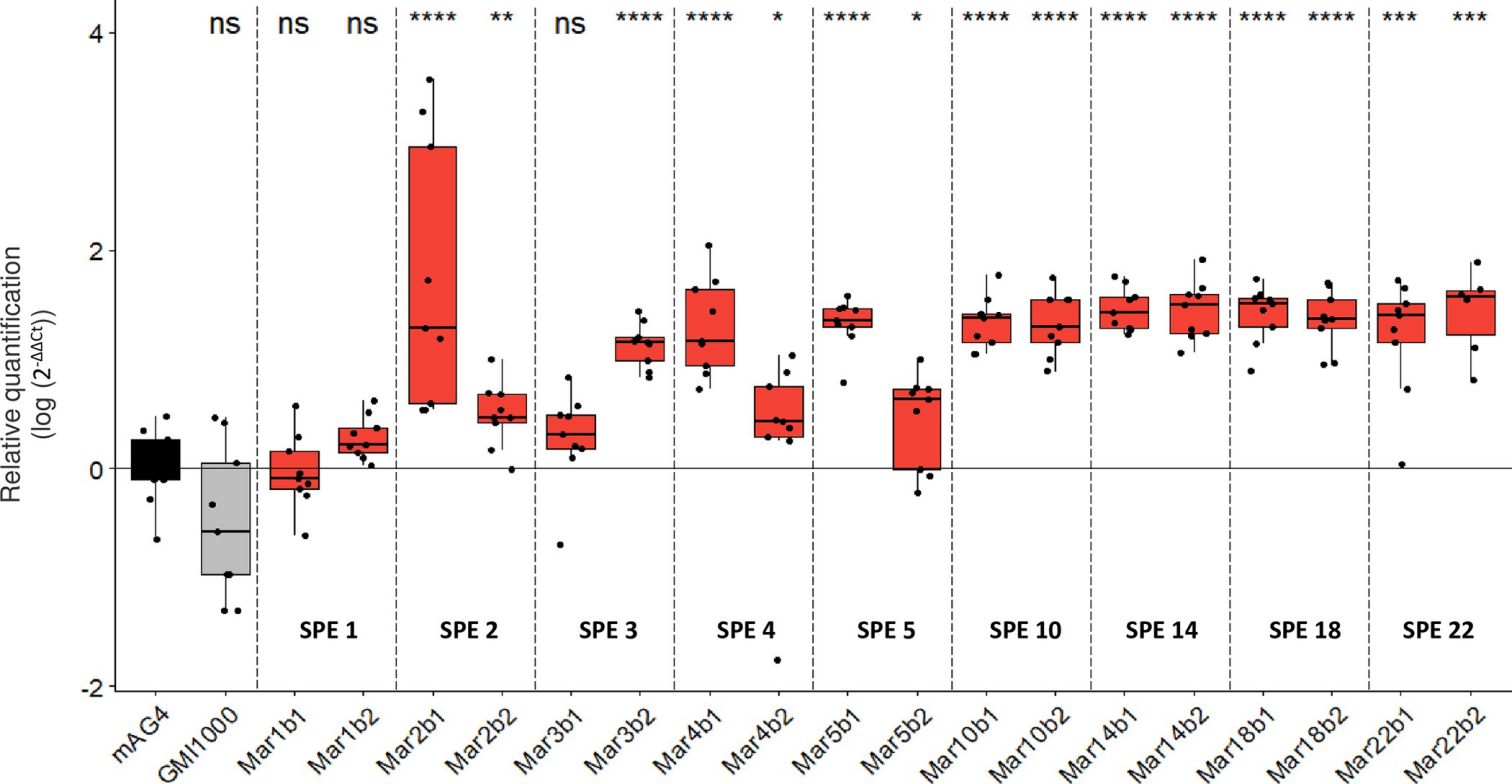
MSRE-qPCR results for chronology of methylation appearance upstream RSp0338 during experimental evolution in tomato Marmande. The methylation profile of the GTWWAC motifs upstream RSp0338 was investigated using a MSRE-qPCR approach for the ancestral GMI1000 clone and the ongoing experimentally evolved clones in tomato Marmande host, lineage B. Evolved clones in tomato Marmande from lineage B were tested at different serial passaging during experimental evolution. Evolved clones were designated with MarXbx notation with X as the number of SPE and x as the clone number. See Figs 5 and 6 for legend. The data underlying this figure can be found in S1 Data.

In this part of the study, we first sought to determine at which stage of evolution the RSp0338 differential methylation occurred. To answer this question, we conducted an MSRE-qPCR analysis on DNA from clones from the tomato Marmande lineage B evolved after 1, 2, 3, 4, 5, 10, 14, 18, and 22 serial passages. Two clones per serial passage were investigated. The MSRE-qPCR results showed that the 2 GTWWAC motifs upstream RSp0338 were already methylated for the 2 clones recovered after 2 passages and remained methylated in all the clones recovered in the following passages (Fig 7).

**Fig 8:**
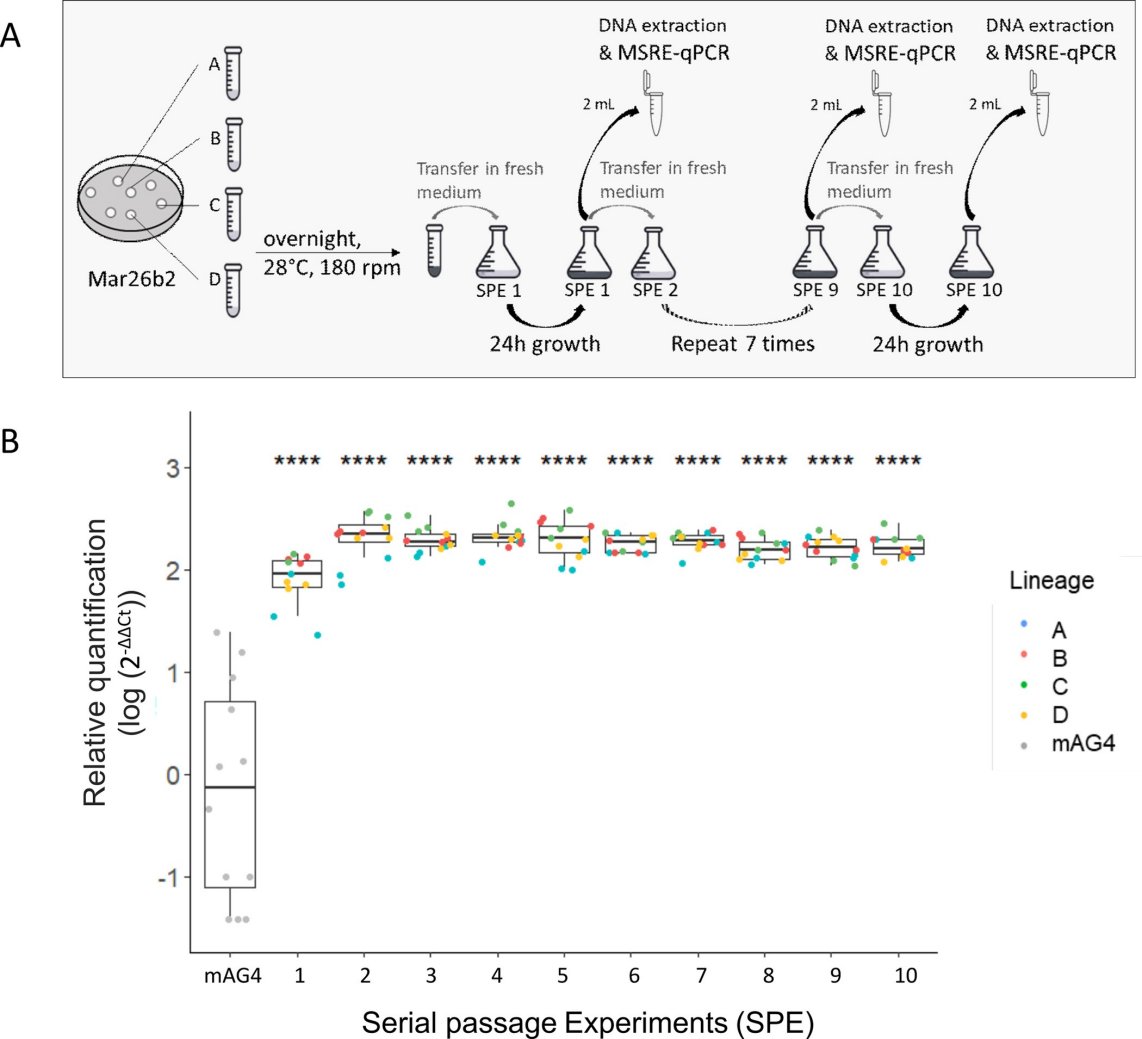
Stability of the methylated state of the GTWWAC motifs upstream RSp0338 in the Mar26b2 evolved clone during the transition from the tomato Marmande environment to the MP glutamine environment. (A) The clone Mar26b2 was serially passaged in synthetic MP medium with glutamine every 24 h. A total of 10 passages were conducted. Around 10 bacterial generations were produced at each passage, thus corresponding to around 100 bacterial generations during the whole experiment. Four biological replicates were conducted, thus generating 4 evolution lineages, named A, B, C, and D. (B) MSRE-qPCR results to evaluate the methylation state upstream RSp0338. The methylation profile of the 2 GTWWAC motifs in the upstream region of the RSp0338 gene in the bacterial population was determined by MSRE-qPCR at each passage. Three technical replicates were conducted per lineage and per passage. See Figs 5 and 6 for legend. The data underlying this figure can be found in S1 Data.

We next attempted to determine to what extent the observed changes in DNA methylation are stable or easily reversed when the growth environment changes. Here, the purpose was to clarify if DNA methylation changes are responsible for long-term adaptation or are rather underlying short-term acclimatization to growth on tomato Marmande. For that purpose, we continued experimental evolution of the Mar26b2 clone, which evolved in tomato Marmande and in which DNA methylation changes were observed, by changing its evolutionary environment. We conducted this experiment in a synthetic medium to move away from the in planta context. Here, the clone Mar26b2 was serially passaged in synthetic MP glutamine medium for a total of 10 passages. The bacteria were recovered after 24 h of growth, and 2.10^7^ bacterial cells were reinoculated in 20 ml of fresh medium at each passage (Fig 8A). An average number of 10 generations was obtained at each passage, thus generating approximately 100 generations after 10 passages. The methylation state upstream RSp0338 was estimated at each passage with DNA extracted from the whole bacterial culture using the MSRE-qPCR approach. The results revealed that the upstream region of RSp0338 is methylated in the first bacterial culture and remains methylated even after 100 generations in synthetic MP glutamine medium (Fig 8B). It is worth noting that the relative MSRE-qPCR quantification is twice as high after 2 passages (20 bacterial generations) than after 1 passage in synthetic MP glutamine medium and then remains stable (Fig 8B). This result could suggest an increase in the population of the frequency of bacterial cells that are methylated in the upstream region of RSp0338 during the first 20 generations until reaching a stable plateau.

### Methylation in the upstream region of RSp0338 contributes to bacterial fitness

In our previous work, we demonstrated that the Mar26b2 clone showed a fitness advantage during growth into the stem of its experimental host, tomato var. Marmande, compared to the ancestral GMI1000 clone, using a competition experiment approach (Table 2) [37].

**Fig 9:**
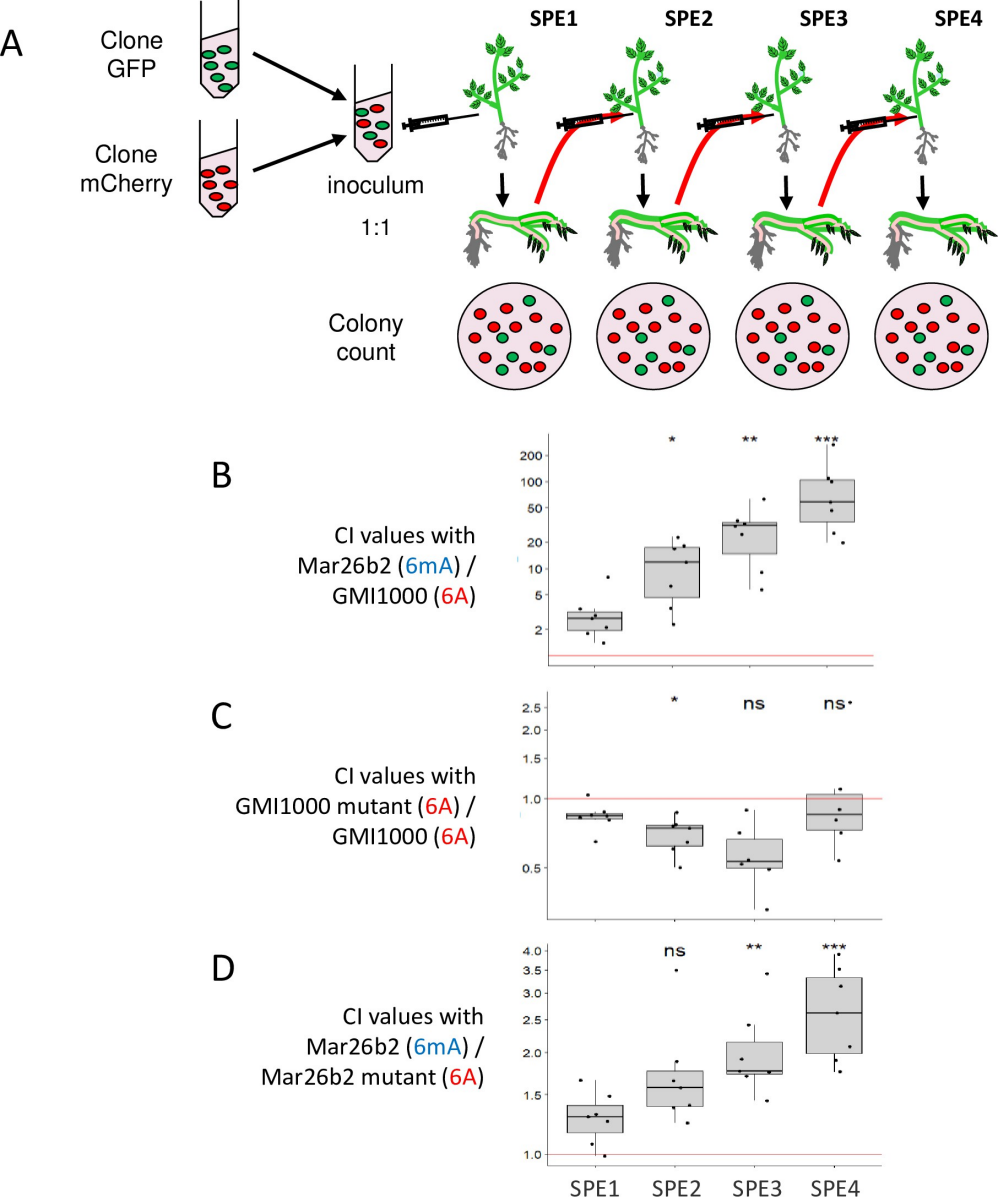
Impact of mutation of the GTWWAC motif in the upstream region of RSp0338 gene on bacterial fitness during growth into tomato var. **Marmande.** (**A**) SPEs were conducted starting with a mixed inoculum of 2 clones, tagged with a GFP or mCherry marker, in the same proportion. At each passage, the CI between the 2 clones was calculated. (**B**) CI values of the Mar26b2 evolved clone in competition with the GMI1000 ancestral clone after 1, 2, 3, and 4 SPE. (**C**) CI values of the GMI1000 mutant in competition with the GMI1000 ancestral clone after 1, 2, 3, and 4 SPE. (**D**) CI values of the Mar26b2 evolved clone in competition with the Mar26b2 mutant after 1, 2, 3, and 4 SPE. In brackets are indicated the methylation profiles of the GTWWAC motifs in the upstream region of the RSp0338 gene for each investigated clone and mutant. The red bar highlights CI = 1. Wilcoxon test, *p-value < 0.05; **p-value < 0.01; ***p-value < 0.001. The data underlying this figure can be found in S1 Data. CI, competitive index; GFP, green fluorescent protein; SPE, serial passage experiment.

In order to analyze the contribution of methylation in the upstream region of the RSp0338 gene in fitness gain of the Mar26b2 clone into tomato var. Marmande, we first constructed mutants of both GMI1000 and Mar26b2 strains in which the 2 GTWWAC motifs in the upstream region of RSp0338 were modified, so that they can no longer be methylated. The GTWWAC motifs modification was performed by introduction of a point mutation replacing the T by a C (Table 8). In a second step, we measured the impact of these mutations on the bacterial fitness into tomato var. Marmande. Our hypothesis was that the strains having a fitness advantage into tomato var. Marmande should enhance their frequency in the population after serial passage experiments (SPEs) in this host. We thus conducted SPE in tomato var. Marmande starting with a mixed inoculum of the investigated clones and mutants and measured the CI after each passage (Fig 9A). Competition SPE with the Mar26b2 and GMI1000 clones validated the fitness advantage of the Mar26b2 clone with CI values enhancing at each passage (Fig 9B). Competition SPE with the GMI1000 mutant and GMI1000 wild-type strain showed that the CI values were not significantly different from one at each passage, thus demonstrating that point mutations of the GTWWAC motifs of the RSp0338 upstream region did not impact the fitness of the GMI1000 strain (Fig 9C). Competition SPE with the Mar26b2 clone and Mar26b2 mutant showed an increase in CI values at each passage (even if this increase was not as high as the increase observed for Mar26b2 and GMI1000 competition), thus demonstrating a fitness advantage of Mar26b2 clone compared to Mar26b2 mutant (Fig 9D). Considering that point mutations of the GTWWAC motifs of the RSp0338 upstream region do not impact the fitness (Fig 9C), these results show a role of methylation of these GTWWAC motifs in adaptive advantage of Mar26b2 clone for growth into the stem of tomato var. Marmande.

**Table 8:** Methylation profiles of GMI1000 and Mar26b2 clones and their corresponding RSp0338-GTWWAC mutants at the beginning of the stationary phase during growth in synthetic medium with glutamine.

Gene ID	Motifs	Mutations	DNA strand	GMI1000	GM1000 mutant	Mar26b2	Mar26b2 mutant
*RSp0338*	GTAAACAAAAAGGTTTAC	G**C**AAACAAAAAGG**C**TTAC	+	6A6A	6A6A	6mA6mA	6A6A
	CATTTGTTTTTCCAAATG	C**G**TTTGTTTTTCC**G**AATG	−	6A6A	6A6A	6mA6mA	6A6A

Note: The point mutations are underlined.

## Discussion

### DNA methylation changes during experimental adaptation of *R*. *pseudosolanacearum* to multihost species

In our previous works, multihost experimental evolution of the GMI1000 strain of *R*. *pseudosolanacearum* selected for clones with a better fitness but little or even no genetic changes [37,39]. We also observed important transcriptomic variations in these evolved clones even in the clones with no mutation [39,42]. These observations led us to propose the hypothesis of a role of epigenetic processes in host adaptation. In the present work, we tested this hypothesis by analyzing 1 epigenetic process, the adenine methylation. We thus investigated 6mA methylation pattern of the ancestral GMI1000 clone and 31 experimentally evolved clones using SMRT-seq technology. This analysis identified a list of 50 putative DMSs at the GTWWAC motif with a varying number of 12 to 21 DMSs per evolved clone. This list included 30 differential hemimethylated (1 DNA strand) and 10 DMSs (both DNA strands). In bacteria, hemimethylated DNA is produced at every round of DNA replication. This DNA modification is generally transient because the DNA MTases quickly re-methylate the majority of their target motifs. However, stable hemimethylated and unmethylated motifs have been reported in various organisms including bacteria [22,25,51]. This phenomenon is well documented in *Escherichia coli* and *Salmonella* Typhimurium where stable hemimethylated and unmethylated GATC sites are formed when a DNA- binding protein protects hemimethylated DNA from Dam methylase activity [22]. Differential methylation pattern on the DNA are involved in phenotypic variation by impacting gene expression through the differential affinity of some transcription factors for methylated versus unmethylated or hemimethylated promoters [52].

The MSRE-qPCR approach was used as an alternative method to investigate the methylation state of the 10 two-strand-DMSs detected by SMRT-seq. MSRE-qPCR appeared to be more stringent, founding only a small proportion of the DMSs detected by SMRT-seq. Only 1 site, upstream of the RSp0338 gene, was detected between the ancestral clone and 3 evolved clones to be differentially methylated by using the MSRE-qPCR approach. A technical reason may explain this discrepancy, because restriction endonuclease sensitive to methylation can display various rates of cleavage depending on several parameters (time of digestion, amount of enzyme, flanking sequence…) and therefore do not always cut 100% of the DNA motifs they recognize [53]. Another possible reason for this discrepancy could be dependent on phenotypic heterogeneity, which is common in bacterial populations [52]. This phenomenon has already been observed in populations of *R*. *pseudosolanacearum* GMI1000 [41]. Several mechanisms involved in the generation of phenotypic heterogeneity include epigenetic regulations [52,54]. This could explain why different methylation states were found using either the SMRT-seq or MSRE-qPCR technologies. It should be remembered that MSRE-qPCR can only detect two-strand methylated sites, unlike SMRT-seq, but it is likely that both methods generate false positives and false negatives. Nevertheless, SMRT-seq already provides a first comprehensive view of 6mA methylation profile of both ancestral and evolved clones, and the combination of the 2 methods has enabled us to robustly validate 2 DMSs upstream RSp0338 between the ancestral and 3 evolved clones.

### Methylation changes at a specific gene rarely correlate with changes in its expression

Among the 31 investigated evolved clones, 39 genes had a potential DMS mark. The analysis of the association between differential methylation and differential expression of a specific gene, however, did not find any correlation except for the RSp0338 gene. These data support a recent analysis of the *S*. Typhimurium methylome and transcriptome, showing that DNA methylation changes generally do not correlate with obvious changes in expression of the differentially methylated gene [55]. Here again, however, phenotypic heterogeneity within bacterial populations could also be another possible reason for the observation of a lack of correlation between the differential methylation of a locus and its differential expression. Adenine methylation in *S*. *enterica* ser. Typhimurium was recently reported to control heterogeneous expression of 7 loci and the formation of many cells in ON or OFF transcriptional states within the same bacterial culture [56]. Nevertheless, comparison of the numbers of differentially methylated genes and differentially expressed genes in each of the evolved clones showed a positive correlation. This suggested that adenine methylation might be involved to some extent in gene expression regulation, even indirectly. However, it is certainly not the only process causing the significant transcriptomic variations observed in the evolved clones and reported in our previous works [42] For example, cytosine methylation or other epigenetic processes could also correlate with the observed transcriptomic variations.

Concerning the RSp0338 gene, the 2 GTWWAC motifs that were detected as differentially methylated are located 321 bp and 309 bp upstream the start codon, thus potentially affecting the promoter region. The correlation between differential methylation and differential expression of the RSp0338 gene suggested an epigenetic regulation, a phenomenon reported in prokaryotes, although still scarcely investigated [45]. Epigenetic regulation in bacteria was reported to result from the impact of DNA methylation on the interaction of DNA-binding proteins with their cognate sites or on changes in DNA topology [22,52,57]. Here, we provide evidence that RSp0338 is a novel example of epigenetically regulated gene in bacteria.

### Why adapt through methylation?

Epigenetic mutations are known to occur at a faster rate than genetic mutation [9,58]. The novel methylation state of the RSp0338 promoter appeared very quickly in the experimental evolution since it was detected from the first 2 serial passages on the host plant. We hypothesize that such fast epigenetic changes can allow rapid adaptation to new environmental conditions. There is also the plausibility that epimutation is easier to generate (and especially to revert) than a genetic mutation and that this property is therefore favorable to rapid adaptation in fluctuating environments.

A major question concerned the stability of the novel methylation profile and how it will influence long-term adaptation to new environments. More and more studies report the existence of stable “epialleles” that are transmitted intergenerationally and affect the phenotype of offsprings. In the same way as conventional DNA sequence-based alleles, these epialleles could be subjected to natural selection, thus contributing to long-term evolutionary processes [59]. Other studies support the hypothesis of the genetic assimilation theory by which epigenetic changes could facilitate genetic mutation assimilation [5,10–13,60]. Here, we addressed the question of the stability of the observed changes in DNA methylation by considering the life cycle of the pathogen that alternates between 2 different environments, inside and outside the plant. We demonstrated that when the bacteria, in which the DNA methylation change was observed, was removed from the plant, the methylation change was maintained, at least for 100 generations outside the host. This analysis prompts us to suggest that the observed change in DNA methylation was fixed in the bacteria and thus can contribute to long-term adaptation rather than short-term acclimatization to growth inside the plant.

### Evidence that methylation changes in RSp0338 (*epsR*) provides adaptation

Using a site-directed mutagenesis approach targeting the 2 GTWWAC motifs upstream RSp0338, we prevented the methylation by the MTase of these 2 motifs in the mutated evolved clone. An in planta competition experiment between the mutant and the evolved clone demonstrated that methylation of the motifs in the upstream region of the RSp0338 gene gives an adaptive advantage. To our knowledge, this is the first study showing a direct link between bacterial epigenetic variation and adaptation to a new environment. The involvement of epigenetic variation in environmental adaptation has been reported in several eukaryotic species [8,61]. In bacteria, the role of epigenetic mechanisms was also reported in antibiotic resistance [62,63]. Here, we found evidence that methylation in the upstream region of RSp0338 provides adaptation, although the adaptive gain of the evolved clone versus mutated clone is not as strong as that of the evolved clone versus ancestral clone (Fig 9). It is therefore likely that the adaptive gain of the evolved clone would also result from the contribution of additive genetic modifications or epigenetic processes other than adenine methylation. The RSp0338 gene has been characterized in the past as *epsR* [47], but its function remains unclear. EpsR, a putative DNA-binding protein, was shown to regulate EPS production in the *R*. *solanacearum* species complex since its overproduction strongly represses EPS synthesis but inactivation of the gene did not obviously affect EPS production [47,64]. Based on this knowledge, it is difficult to infer a role for the decrease in *epsR* expression (as suggested by the transcriptomic data from evolved clones) linked to methylation of its promoter. Nevertheless, it is certain that *epsR* is, directly or indirectly, linked to the PhcA-dependent virulence regulation network in *R*. *solanacearum* [64,65] and probably contributes to the control of EPS production or associated molecules. It should be noted that during the evolution of GMI1000 by serial passages on several host plants, alterations in another regulatory gene, *efpR*, conferring strong adaptive gains were selected and lead to multiple phenotypic changes, including significant modifications for EPS production [40,41]. We can therefore hypothesize that the production of these surface/excreted molecules plays an important role in the phases of adaptation to the environmental conditions encountered during plant infection, and future work will need to establish their role at this level. In conclusion, our identification of a differential DNA methylation mark involved in adaptation of a plant pathogen to its host emphasizes the importance of considering the role of any possible bacterial epigenetic mechanisms in adaptation to new environments in future studies.

## Materials and methods

### Bacterial strains, plant material, and growth conditions

The GMI1000 strain and the 31 derived evolved clones investigated in this study are described in Table 2. The evolved clones generated after experimental evolution include 10 clones evolved in tomato Hawaii 7996 (*Solanum lycopersicum*) [39], 7 clones in eggplant MM61 (*S*. *melongena* var. Zebrina), 3 clones in bean (*Phaseolus vulgaris* var. Blanc Précoce), 6 clones in tomato Marmande (*S*. *lycopersicum* var. Super Marmande), and 5 clones in cabbage (*Brassica oleracea* var. Bartolo) [37]. The bacterial strains were grown at 28°C (under agitation at 180 rpm for liquid cultures) either in BG complete medium or in MP synthetic medium [66]. The pH of the MP medium was adjusted to 6.5 with KOH. For agar plates, BG medium was supplemented with D-Glucose (5 g/l) and triphenyltetrazolium chloride (0.05 g/l). The MP medium was supplemented with L-Glutamine (10 mM) and oligo elements (1,000 mg/l) [42].

Four- to 5-wk-old tomato (*Solanum lycopersicum*) cultivar Marmande plants were used for the in planta bacterial competition assays. Tomato plants were grown in a greenhouse. In planta competition experiments were conducted in a growth chamber under the following conditions: 12-h light at 28°C, 12-h darkness at 27°C and 75% humidity.

### SMRT-seq

Genomic DNA was prepared from the bacterial cells grown in synthetic media with glutamine collected at the beginning of stationary phase in order to limit the number of cells in division and avoid a bias towards hemimethylated marks. The bacterial samples were collected as described previously [39]. Briefly, each of the evolved clones and the ancestral clone GMI1000 were grown in MP medium with 10 mM glutamine. For whole genome sequencing, 20 ml of the bacterial culture was centrifuged at 5,000*g* for 10 min followed by washing the pellets with water and centrifuged again. The pellets were stored at −80°C until DNA extraction. The DNA were prepared based on the protocol described for high molecular weight genomic DNA [67]. Library preparation was performed at GeT-PlaGe core facility, INRAE Toulouse, France, and SMRT sequencing at Gentyane core facility, INRAE Clermont-Ferrand, France. Eight libraries of multiplex samples were performed according to the manufacturer’s instructions “Procedure-Checklist-Preparing-Multiplexed-Microbial-SMRTbell-Libraries-for-the-PacBio-Sequel-System.” At each step, DNA was quantified using the Qubit dsDNA HS Assay Kit (Life Technologies), and DNA purity was tested using a NanoDrop spectrophotometer (Thermo Fisher Scientific). Size distribution and degradation were assessed using the Fragment analyzer (Agilent) and High Sensitivity Large Fragment 50 kb Analysis Kit (Agilent). Purification steps were performed using AMPure PB beads (PacBio). The 32 individual samples (2 μg) were purified and then sheared at 10 kb using the Megaruptor1 system (Diagenode). Using SMRTBell template Prep Kit 1.0 and SMRTbell Barcoded Adaptater kit 8A or 8B kits (PacBio), samples (1 μg) were independently barcoded and then pooled by 5 to 8. The 8 libraries were purified 3 times. SMRTbell libraries were sequenced on SMRTcells on Sequel1 instrument at 6pM with 120-min preextension and 10-h or 20-h movies using Sequencing Primer V4, polymerase V3, diffusion loading.

### GTWWAC methylation analysis

All methylation analyses were performed with public GMI1000 genome and annotation. Motif and methylation detection were performed using the pipeline “pbsmrtpipe.pipelines.ds_modification_motif_analysis” from PacBio SMRTLink 6.0. The default settings were used except compute methyl fraction set as true, minimum required alignment concordance > = 80, and minimum required alignment length > = 1,000.

Followed by the bioinformatics analyses of the data obtained from SMRT-seq, methylome profiles of the 31 evolved clones were compared to the ancestral clone individually. The analysis showed the methylation profile for G**T**WWAC motif with a score, coverage, IPD ratio, and fraction for each sample. A score above 30 is considered significant, and coverage represents the sequencing depth (higher the better). IPD ratio or interpulse duration ratio is the time required for the consequent nucleotide to bind, where the presence of methylated base increases the time required for the nucleotide addition (higher IPD ratio means a higher probability of methylation). The fraction represents the percentage of methylated bases in the genome pool at that particular position. In this experiment, the methylation or hemimethylation of a particular position is considered significant when the fraction is greater than or equal to 0.50 (represents at least 50% of the sequences are methylated at that particular position in the whole genome pool) in addition to the score above 30.

The correlation between the number of DMSs and DEGs in each evolved clone was estimated by calculating a Spearman’s rank correlation coefficient (rho) using the cor.test function from the stats R package and the R software. The correlation between differential methylation and differential gene expression of the corresponding gene was estimated by conducting a Fisher’s exact test using the fisher.test function from the stats R package and the R software.

### MSRE-qPCR

The MSRE-qPCR approach was used to check the methylation profile at a specific genomic region [50]. The protocol used for MSRE-qPCR derived from Payelleville and colleagues [17]. Genomic DNA was extracted from bacterial cells grown in the same culture condition (synthetic MP medium with glutamine) and at the same growth stage (beginning of stationary phase) used for SMRT-seq. Genomic DNA extraction and purification was performed using the Genomic DNA Purification Kit from Promega. First, in order to generate numerous linear DNA fragments, 400 ng of genomic DNA was digested by *Eco*RI (0.25 U in a total volume of 20 μL) for 1 night at 37°C followed by an enzyme inactivation step (20 min at 65°C). Then, 8 μl of *Eco*RI-digested-DNA was digested by *Hpy166*II (0.25 U in a total volume of 20 μL) for 1 night at 37°C followed by an enzyme inactivation step (20 min at 65°C). The *Hpy166*II restriction enzyme digests only unmethylated GTNNAC sites. A qPCR amplification was then performed on 2 μl of 10^−4^ diluted DNA in a total volume of 7 μl containing 3.6 μl of Master mix Takyon SYBR Green I and 0.5 mM of each primer. Primers used for MSRE-qPCR are described in S3 Table. The qPCR amplification was performed using the LightCycler 480 II (Roche) and the following program; 3 min of Takyon activation at 95°C, and 45 cycles of denaturation 10 s at 95°C and primer annealing/extension 45 s at 65°C. Detection of an amplicon revealed that no digestion occurred and that the region was methylated, while non-amplification revealed that the region was unmethylated and digested. The mAG4 mutant (GMI1000 deleted from the RSc1982 MTase, targeting GTWWAC motifs; see mutant construction below) was used as a nonmethylated control at GTWWAC motifs (a negative control for qPCR amplification). *Eco*RI digested DNA diluted 10^−4^ times was used as a positive control for qPCR amplification.

Raw data from qPCR experiments were analyzed using the 2^−ΔΔCt^ method to perform a relative quantification [49]. This method was used to relate the PCR signal of the MSRE digested DNA to the PCR signal of the *EcoR*I digested DNA. Ct values obtained with MSRE-digested DNA were first normalized with mean Ct value obtained with *EcoR*I-digested DNA (ΔCt = CtMSRE-DNA−meanCt_EcoRI-DNA_). This ΔCt value was then normalized with the mean ΔCt value obtained with mAG4 DNA to calculate the ΔΔCt value ΔΔCt = ΔCtevolved.clone−meanΔCt_mAG4_). The amplification efficiency of the target and reference primers were checked and close to 2. Therefore, the amount of target, normalized to the reference and relative to the calibrator, was given by the 2^−ΔΔCt^ value [49]. Three biological replicates and 3 to 6 technical replicates were performed. The 2^−ΔΔCt^ values were compared using the Wilcoxon nonparametric test with the R software.

### Experimental evolution in synthetic medium

The clone Mar26b2 was revived from glycerol stock on plates containing BG complete medium for 2 d at 28°C. One individual colony was used to inoculate a 15-ml test tube containing 5-mL synthetic MP-glutamine liquid medium and was incubated overnight at 28°C under agitation at 180 rpm. After 24 h, optical density at 600 nm (OD_600_) was measured, and the preculture was used to inoculate a 100-mL Erlenmeyer flasks containing 20-mL MP-glutamine at a starting OD_600_ = 0.001. After 24 h of growth at 28°C under agitation at 180 rpm, OD_600_ was measured, and the culture was used to reinoculate a new 100-mL Erlenmeyer flasks containing 20-mL MP-glutamine at a starting OD_600_ = 0.001. A 1-ml aliquot of the culture was stored at −80°C in the presence of 20% glycerol. Another aliquot of 2 ml of culture was centrifuged for 2 min at 13,000 rpm and stored at −80°C until DNA extraction for the MSRE-qPCR analysis. The same protocol was applied 10 times, thus representing 10 serial passages in synthetic MP glutamine medium (Fig 8A). Four biological replicates were conducted, thus generating 4 evolutionary lineages, named A, B, C, and D.

### Construction of mutants

The GMI1000 mAG4 unmarked deletion mutant (gene RSc1982) was constructed using *sacB* counterselectable marker as described in Gopalan-Nair and colleagues [39]. Briefly, 2 border fragments of the RSc1982 gene were PCR-amplified using primers with flanking restriction sites, *EcoR*I and *Xba*I for the upstream fragment, *Xba*I and *Hind*III for the downstream fragment (S3 Table). These 2 fragments were ligated and cloned in the pK18 plasmid [68]. This construction was used to transform GMI1000 competent cells as described in Perrier and colleagues [69]. The gene deletion was checked by PCR on colonies, after selection of those that were resistant to sucrose and sensitive to kanamycin.

Point mutations of the 2 GTWWAC motifs, changing a T by a C, upstream the RSp0338 gene were conducted on both the ancestral GMI1000 clone and the Mar26b2 evolved clone. These point mutations were performed using primers carrying the desired mutations (RSp0338_R1_CC and RSp0338_F2_CC; S3 Table) and cloning primers with flanking *EcoR*I and *Hind*III restriction sites (RSp0338_F1 and RSp0338 R2; S3 Table). Two PCRs were performed on GMI1000 or Mar26b2 genomic DNA, PCR1 with the RSp0338_F1 and RSp0338_R1_CC primers and PCR2 with the RSp0338_F2_CC and RSp0338_R2 primers. These 2 PCR products were then mixed and used as a matrix for a third PCR reaction with RSp0338_F1 and RSp0338_R2 primers (fusion PCR by overlap between DNA fragments 1 and 2). The obtained PCR product was then cloned in a pK18 plasmid, and this construction was used to transform GMI1000 or Mar26b2 competent cells as described in Perrier and colleagues [69]. The point mutations were checked by PCR on the colonies that were resistant to sucrose and sensitive to kanamycin, followed by Sanger sequencing. All mutants were tagged with the fluorescent reporters mCherry or GFP as previously described [41]. All primers used for the construction of mutants are listed in S3 Table.

### RT-qPCR analysis

The RT-qPCR approach was used to quantify the expression of the RSp0338 gene in the ancestral GMI1000 clone and the evolved clones. The protocol used for RT-qPCR derived from Perrier and colleagues [40]. Total RNAs were isolated using TRIzol Reagent (Life Technologies) followed by RNeasy MiniElute Cleanup Kit (Qiagen). To avoid contamination by genomic DNA, each sample was treated with the TURBO DNA-free Kit (Life Technologies). The reverse transcription was performed on 1 μg of total RNA using the Transcriptor Reverse Transcriptase (Roche) with random hexanucleotides primers. Quantitative PCRs were performed on a Roche LightCycler480 as described for MSRE-qPCR. The specificity of each amplicon was validated with a fusion cycle. The efficiency of amplification was tested with dilution game and calculated using −1+10^1/slope^ formula. The expression of RSp0338 was normalized using the geometric average of 3 selected reference genes (RSc0403, RSc0368, and RSp0272) for each sample and calculated using the 2^−ΔΔCt^ method [40,49,70,71]. All kit and reagents were used following the manufacturer’s recommendations. The primer sets used in the experiments are listed in S3 Table.

### Bacterial competition assay and in planta serial passage experiments

The bacterial competitive assay was performed as previously described [41]. Briefly, 10 μl of the mixed inoculum, containing the GFP and mCherry clones in equal proportion at a 10^6^ CFU/ml concentration, was injected into the stem of tomato cv. Marmande, 1 cm above the cotyledons. Bacteria were recovered from the plant stem as soon as the first wilting symptoms appeared (3 to 5 d after inoculation) as previously described [37].

Four SPEs into the stem of tomato cv. Marmande were performed. At each SPE, serial dilutions of the recovered bacterial suspension were conducted. Around 10 μl of the 10^−3^ dilution was directly injected into the stem of a healthy plant, and 50 μl of the 10^−4^ and 10^−6^ dilutions were plated on BG complete medium without triphenyltetrazolium chloride using an automatic spiral plater (easySpiral, Interscience, France). Green and red colonies were visualized and enumerated using a fluorescence stereo zoom microscope (Axio Zoom.V16, ZEISS, Germany). A CI was calculated at each SPE as the ratio of the 2 clones obtained from the plant stem (output) divided by the ratio in the inoculum (input) [72]. A total of 7 replicates were performed for each competition assay. Differences between mean CI values were tested using a Wilcoxon test performed in the R statistical software.

## Supporting information

S1 Fig Effect of the experimental host on the number of DMSs detected in the evolved clones according to SMRT-seq. (**A**) Number of DMSs in each investigated evolved clone. (**B**) Mean number of DMSs in evolved clones for each experimental host. Different letters above the boxplot indicate a significant difference (Wilcoxon test, *p*-value < 0.05). Mar: Tomato var. Marmande; Zeb: Eggplant var. Zebrina; Bean: Bean var. Blanc précoce; Cab: Cabbage var. Bartolo; Haw: Tomato var. Hawaii 7996. The data underlying this figure can be found in S1 Data.
(PPTX)

S2 Fig Correlation between the number of mutation and the number of DMSs in each evolved clone. (**A**) Spearman correlation coefficient was calculated and is indicated. The data underlying this figure can be found in S1 Data.
(PPTX)

S1 Table Genomic regions of the GMI1000 strain of *R*. *pseudosolanacearum* with a GTWWAC motif and methylation status at the beginning of the stationary phase during growth in synthetic medium with glutamine 10 mM for the ancestral clone and the 31 experimentally evolved clones, according to SMRT-seq data analysis. *GTWWAC motifs were annotated intragenic if their positions mapped within the annotated coding sequence, upstream if they mapped to the first noncoding 300 bp before the annotated start codon and downstream otherwise. **For hemimethylated motifs, the strand that is methylated is indicated.
(XLSX)

S2 Table **(a) Correlation between methylation fractions and gene expression levels as estimated by the Spearman correlation coefficient. (b) Methylation mean fraction according to SMRT-seq data and RNAseq counts for each gene in the ancestral GMI1000 strain and the 31 evolved clones grown in synthetic medium supplemented with glutamine and collected at the beginning of stationary phase** [42].
(XLSX)

S3 Table List of primers used in this study. (XLSX)

S1 Data Supporting information underlying Figs 1, 3, 4, 5, 6, 7, 8, 9, S1, and S2. (XLSX)

## Abbreviations

CI: competitive index
DEG: differentially expressed gene
DMS: differential methylated site
EPS: exopolysaccharide
MSRE-qPCR: methylation-sensitive restriction enzyme-quantitative PCR
MTase: methyltransferase
RSSC: *Ralstonia solanacearum* species complex
RT-qPCR: quantitative reverse transcription PCR
SMRT-seq: sequencing of single molecules in real time
SPE: serial passage experiment
TE: transposable element
4mC: 4-methylcytosine
5mC: 5-methylcytosine
6mA: 6-methyladenine
