# Supplementary material for: Changes in DNA methylation contribute to rapid adaptation in bacterial plant pathogen evolution
Source: PLoS Biol. 2024 Sep 20;22(9):e3002792. doi: 10.1371/journal.pbio.3002792 (PMC11460718; doi:10.1371/journal.pbio.3002792)
Supplement: S1 Fig — (A) Number of DMSs in each investigated evolved clone. (B) Mean number of DMSs in evolved clones for each experimental host. Different letters above the boxplot indicate a significant difference (Wilcoxon test, p-value < 0.05). Mar: Tomato var. Marmande; Zeb: Eggplant var. Zebrina; Bean: Bean var. Blanc précoce; Cab: Cabbage var. Bartolo; Haw: Tomato var. Hawaii 7996. The data underlying this figure can be found in S1 Data. (PPTX) [file pbio.3002792.s001.pptx]

## Slide 1
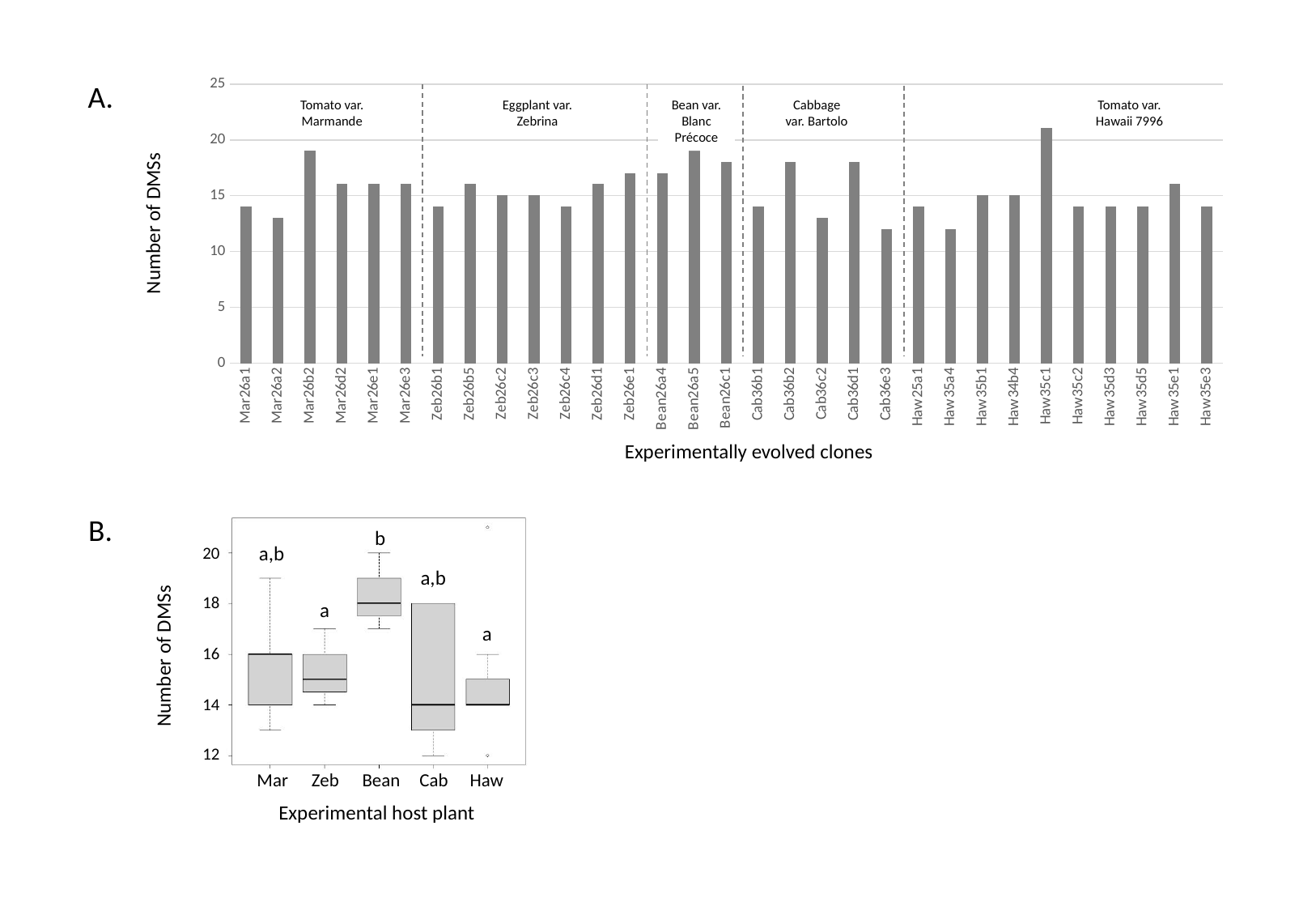

### Chart
| Category | nb DMR |
|---|---|
| Mar26a1 | 14.0 |
| Mar26a2 | 13.0 |
| Mar26b2 | 19.0 |
| Mar26d2 | 16.0 |
| Mar26e1 | 16.0 |
| Mar26e3 | 16.0 |
| Zeb26b1 | 14.0 |
| Zeb26b5 | 16.0 |
| Zeb26c2 | 15.0 |
| Zeb26c3 | 15.0 |
| Zeb26c4 | 14.0 |
| Zeb26d1 | 16.0 |
| Zeb26e1 | 17.0 |
| Bean26a4 | 17.0 |
| Bean26a5 | 20.0 |
| Bean26c1 | 18.0 |
| Cab36b1 | 14.0 |
| Cab36b2 | 18.0 |
| Cab36c2 | 13.0 |
| Cab36d1 | 18.0 |
| Cab36e3 | 12.0 |
| Haw25a1 | 14.0 |
| Haw35a4 | 12.0 |
| Haw35b1 | 15.0 |
| Haw34b4 | 15.0 |
| Haw35c1 | 21.0 |
| Haw35c2 | 14.0 |
| Haw35d3 | 14.0 |
| Haw35d5 | 14.0 |
| Haw35e1 | 16.0 |
| Haw35e3 | 14.0 |A.
Tomato var. Marmande
Eggplant var. Zebrina
Bean var. Blanc Précoce
Cabbage var. Bartolo
Tomato var. Hawaii 7996
Number of DMSs
Experimentally evolved clones
B.
b
a,b
20
a,b
18
a
a
16
Number of DMSs
14
12
Mar
Zeb
Bean
Cab
Haw
Experimental host plant
