# Supplementary material for: Changes in DNA methylation contribute to rapid adaptation in bacterial plant pathogen evolution
Source: PLoS Biol. 2024 Sep 20;22(9):e3002792. doi: 10.1371/journal.pbio.3002792 (PMC11460718; doi:10.1371/journal.pbio.3002792)
Supplement: S2 Fig — (A) Spearman correlation coefficient was calculated and is indicated. The data underlying this figure can be found in S1 Data. (PPTX) [file pbio.3002792.s002.pptx]

## Slide 1
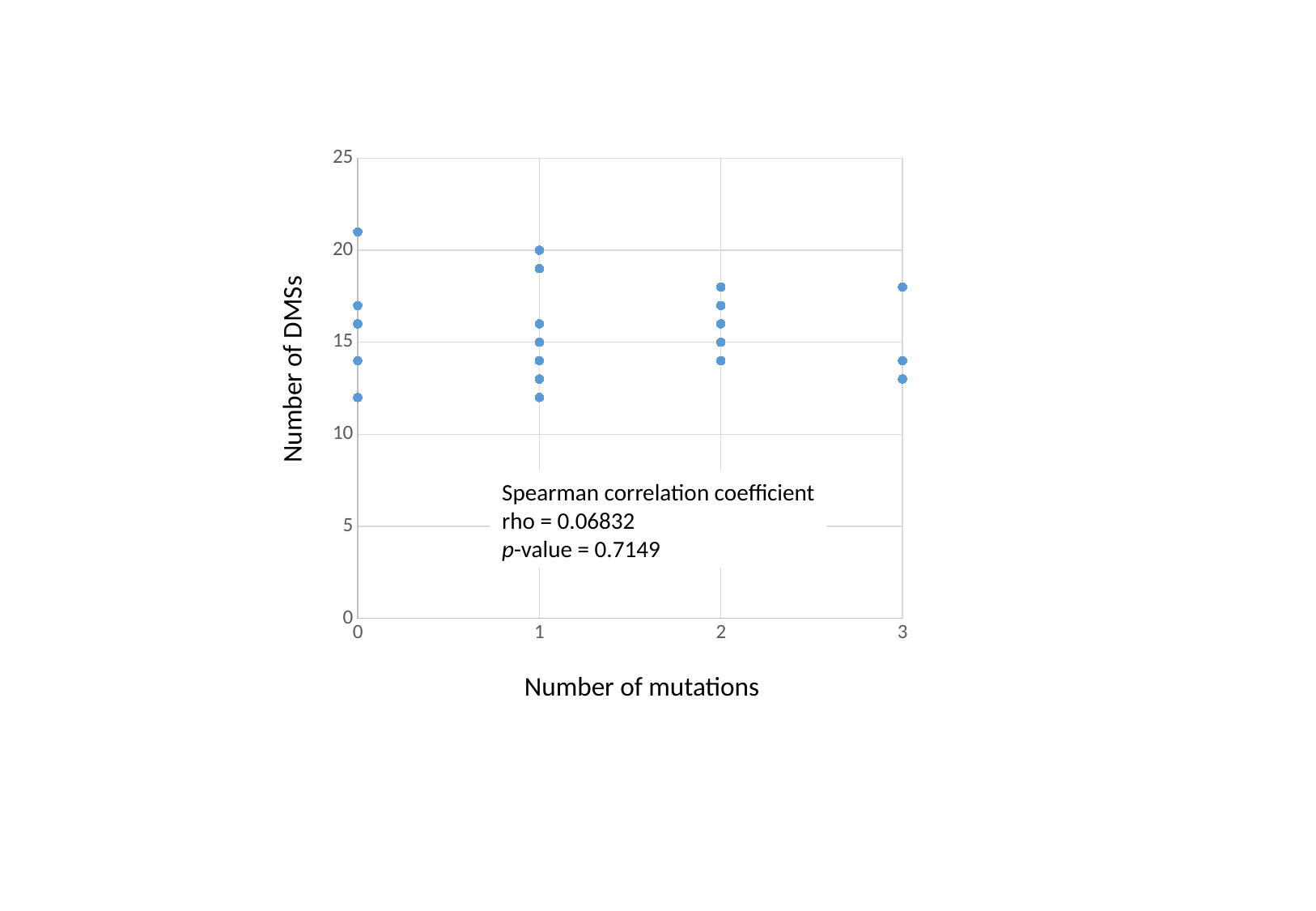

### Chart
| Category | nb DMR |
|---|---|Number of DMSs
Spearman correlation coefficient
rho = 0.06832
p-value = 0.7149
Number of mutations
